# Estimated impact on birth weight of scaling up intermittent preventive treatment of malaria in pregnancy given sulphadoxine-pyrimethamine resistance in Africa: A mathematical model

**DOI:** 10.1371/journal.pmed.1002243

**Published:** 2017-02-28

**Authors:** Patrick G. T. Walker, Jessica Floyd, Feiko ter Kuile, Matt Cairns

**Affiliations:** 1 MRC Centre for Outbreak Analysis and Modelling, Department of Infectious Disease Epidemiology, Imperial College London, London, United Kingdom; 2 Malaria Epidemiology Unit, Department of Clinical Sciences, Liverpool School of Tropical Medicine, Liverpool, United Kingdom; 3 Kenya Medical Research Institute, University of Oxford–Wellcome Trust Collaborative Programme, Kenyatta National Hospital, Nairobi, Kenya; 4 MRC Tropical Epidemiology Group, London School of Hygiene & Tropical Medicine, London, United Kingdom; Walter and Eliza Hall Institute of Medical Research, AUSTRALIA

## Abstract

**Background:**

Malaria transmission has declined substantially in the 21st century, but pregnant women in areas of sustained transmission still require protection to prevent the adverse pregnancy and birth outcomes associated with malaria in pregnancy (MiP). A recent call to action has been issued to address the continuing low coverage of intermittent preventive treatment of malaria in pregnancy (IPTp). This call has, however, been questioned by some, in part due to concerns about resistance to sulphadoxine-pyrimethamine (SP), the only drug currently recommended for IPTp.

**Methods and findings:**

Using an existing mathematical model of MiP, we combined estimates of the changing endemicity of malaria across Africa with maps of SP resistance mutations and current coverage of antenatal access and IPTp with SP (IPTp-SP) across Africa. Using estimates of the relationship between SP resistance mutations and the parasitological efficacy of SP during pregnancy, we estimated the varying impact of IPTp-SP across Africa and the incremental value of enhancing IPTp-SP uptake to match current antenatal care (ANC) coverage.

The risks of MiP and malaria-attributable low birthweight (mLBW) in unprotected pregnancies (i.e., those not using insecticide-treated nets [ITNs]) leading to live births fell by 37% (33%–41% 95% credible interval [crI]) and 31% (27%–34% 95% crI), respectively, from 2000 to 2015 across endemic areas in sub-Saharan Africa. However, these gains are fragile, and coverage is far from optimal. In 2015, 9.5 million (8.3 million–10.4 million 95% crI) of 30.6 million pregnancies in these areas would still have been infected with *Plasmodium falciparum* without intervention, leading to 750,000 (390,000–1.1 million 95% crI) mLBW deliveries. In all, 6.6 million (5.6 million–7.3 million 95% crI) of these 9.5 million (69.3%) pregnancies at risk of infection (and 53.4% [16.3 million/30.6 million] of all pregnancies) occurred in settings with near-perfect SP curative efficacy (>99%) based on the most recent estimates of resistance. Forty-four percent of these pregnancies (23% of all pregnancies) were not receiving any IPTp-SP despite making ≥3 ANC visits, representing 160,000 (94,000–236,000 95% crI) preventable low birthweight (LBW) deliveries. Only 4% (1.4 million) of pregnancies occurred in settings with >10% prevalence of the sextuple haplotype associated with compromised SP effectiveness. Forty-two percent of all pregnancies occurred in settings where the quintuple *dhfr/dhps* haplotype had become established but where in vivo efficacy data suggest SP maintains the majority of its effectiveness in clearing infections.

Not accounting for protection from the use of ITNs during pregnancy, expanding IPTp-SP to all women with ≥3 ANC visits in Africa could prevent an additional 215,000 (128,000–318,000 95% crI) LBW deliveries. In 26 countries with sufficient recent data to estimate ITN impact (population-based ITN usage data that can be stratified by gravidity), we estimate that, due primarily to low ITN use by primigravidae, only 16.5% of the potential LBW births prevented by scaling up IPTp-SP would in fact have already have been prevented through ITN use.

Our analysis also highlights the difficulties associated with estimating the relationship between the effectiveness of interventions against parasitological endpoints such as placental infection at delivery and health outcomes including birthweight, which is also determined by a wide range of unrelated factors. We also did not capture other aspects of malaria burden such as clinical malaria, maternal and neonatal anaemia, and miscarriage, all of which increase the overall importance of effective preventative strategies but have their own relationship with transmission intensity, parity, and SP resistance.

**Conclusions:**

Despite recent declines in malaria transmission in Africa, the burden of MiP in the absence of adequate prevention remains substantial. Even accounting for SP resistance, extending IPTp-SP to all women attending ANC, as well as long-lasting insecticidal net distribution targeted towards first-time mothers, would have a sizeable impact upon maternal and infant health in almost all malaria-endemic settings in sub-Saharan Africa.

## Author summary

**Why was this study done?:**

- Universal access to intermittent preventive treatment of malaria in pregnancy with the drug sulphadoxine-pyrimethamine (IPTp-SP) has been a cornerstone of prevention of malaria in pregnancy, and a Global Call to Action has been launched by the Roll Back Malaria Partnership based upon its high safety, acceptability, effectiveness, and cost-effectiveness.
- The development of resistance by the parasite in some regions—particularly a sequential chain of six mutations that may severely compromise the efficacy of the drug—and recent falls in malaria transmission have led some to question the wisdom of this call to action.

**What did the researchers do and find?:**

- Using a mathematical model, we combined maps of the current risk of malaria in pregnancy with maps of the level of drug resistance across Africa to estimate the likely impact of scaling up IPTp-SP, taking into account the effects of resistance.
- We found that in the large majority of pregnancies in areas of sustained malaria transmission in Africa, IPTp-SP is likely to retain substantial effectiveness against malaria in pregnancy, with areas affected by the sextuple mutation representing a small proportion of these pregnancies.
- We found that, even accounting for recent declines in malaria transmission, the benefits of providing IPTp-SP in lots of areas of Africa remain substantial, but uptake of the intervention remains low despite large increases in antenatal clinic attendance in many countries.

**What do these findings mean?:**

- In general, these findings support the suggestion that, if successful, the drive to increase IPTp-SP use will have a substantial and, given that the majority of women already access antenatal care, highly cost-effective impact upon maternal and neonatal health.
- Although the sextuple mutation was confined to only a small proportion of areas in Africa as of 2010, there are large areas of Africa (one in five women of childbearing age at risk reside in such areas) where the quintuple mutation (the immediate precursor to having the sextuple mutation) has reached saturation, which highlights the need for careful monitoring of the spread of resistance and also highlights the benefits of research into alternative strategies if the sextuple mutation spreads.

## Introduction

Malaria in pregnancy (MiP) has long been understood to have a devastating impact on mother and child. Prior to the unprecedented investment in malaria control that has occurred in the 21st century [1], it had been estimated that nearly 20% of low birthweight (LBW) deliveries (35% of those that were preventable) were caused by malaria [2,3], leading to an estimated 11.4% of neonatal deaths and causing 5.7% of infant deaths in malaria-endemic areas of Africa [4]. The prevalence and burden of malaria have declined markedly in the 21st century [5], primarily due to the dramatic increase in the coverage of malaria interventions, with the usage of insecticide-treated nets (ITNs) increasing from <2% in 2000 to 55% in 2015 and the proportion of malaria cases adequately treated increasing from <1% in 2005 to 16% in 2014 [6]. However, in areas of sustained transmission, women of childbearing age infected with malaria parasites remain at high risk of adverse outcomes such as malaria, anaemia, maternal mortality, stillbirth, preterm delivery, and having a LBW baby in the event they become pregnant [7].

Protection from MiP currently relies on provision of long-lasting insecticidal nets (LLINs), prompt management of malaria cases, and intermittent preventive treatment of MiP (IPTp) with sulphadoxine-pyrimethamine (SP). IPTp with SP (IPTp-SP), recommended for all HIV-negative pregnant women living in all areas of moderate to high transmission in Africa, consists of presumptive administration of SP at all scheduled antenatal care (ANC) contacts in the second and third trimester after foetal quickening, given at least 1 mo apart. IPTp-SP has proven to be a safe, well-tolerated [8], efficacious [9–11], and cost-effective [12] means by which to reduce the substantial burden of MiP [13].

However, despite the numerous advantages of IPTp-SP, the provision of this intervention remains low across Africa, with only an estimated 21.5% of women at risk of malaria receiving at least two doses of SP in 2010 where data were available [14]. This persisting low coverage has caused the Roll Back Malaria Partnership of WHO to lead the Global Call to Action, which aims to address the existing barriers to achieving the 2010 scale-up target of 80% IPTp-SP uptake of all pregnant women at risk [15]. This call, and the movement for improving coverage of the intervention in Africa in general, has not, however, been unanimously welcomed [16]. This is in large part due to concerns that SP is a “failing” [17] or “failed” [16] drug in some areas of Africa due to the emergence of parasites with multiple resistance mutations [18].

The efficacy of SP for treating clinical disease [19] and when used for intermittent preventive treatment in infants [20] is substantially inhibited by sequential mutations in the *Plasmodium falciparum dihydrofolate reductase (dhfr)* and *dihydropteroate synthase (dhps)* genes, which confer resistance to pyrimethamine and sulphadoxine, respectively [21]. A recent analysis of the in vivo efficacy of SP to clear existing infections in asymptomatic parasitaemic pregnant women who received SP for IPTp showed that the efficacy of SP is retained both where the *dhfr*-51,59,108 triple mutation is found at high prevalence and where there is a high prevalence of the quadruple mutant (triple *dhfr* mutation and single mutation at *dhps*-A437G) [22]. In vivo efficacy of SP is lower in areas with a high frequency of the quintuple mutant (with an additional *dhps*-K540E mutation). This lower efficacy reflects both the reduced ability of SP to clear existing infections and a reduction in the ability to prevent new infections from occurring, shown by the progressive shortening of the duration of post-treatment prophylaxis relative to areas with low SP resistance [22]. Although representing fewer areas of Africa, settings where the sextuple resistance haplotype is present (with an additional *dhps*-A581G mutation) are of even greater concern, with observational studies showing no effect of SP upon malaria infection and parasite densities [23,24] and one (also observational) study suggesting that SP may actually cause harm in such settings [25].

The aim of this analysis is to estimate the current impact of IPTp-SP taking the following into account: the intrinsic malaria risk and associated LBW burden (i.e., that which would occur in the absence of protection from either ITNs or IPTp), the most recent estimates of coverage of interventions against MiP (IPTp-SP and ITNs), geographic patterns of SP resistance, and the effect of SP resistance on IPTp-SP efficacy. We also explore the potential impact of IPTp-SP if coverage was fully scaled up to match current ANC coverage. This is done in several stages, as follows.

We use recently published estimates of changes in malaria transmission between 2000 and 2015 to assess how the intrinsic MiP risk and LBW burden have changed, and the extent to which they remain an urgent public health issue in Africa. Although LBW is not the only adverse outcome caused by MiP, LBW has a substantial negative impact on those who experience it, leading to increased risk of neonatal and infant mortality and a range of poor developmental outcomes [7]. The disability-adjusted life years associated with LBW have ranged between 8.61 and 23.62 [26–28], and LBW is generally on the causal pathway of most of the burden calculated in cost-effectiveness analyses of MiP interventions, with the impact on LBW a primary driver of the estimated high cost-effectiveness of IPTp-SP [12,26–28].

To assess the adequacy of current preventative measures to prevent this burden, we then combine our up-to-date estimates of the number of pregnancies at risk of malaria and malaria-attributable LBW (mLBW) with the most recent estimates of the coverage of IPTp-SP and use of ITNs in pregnancy.

In order to assess the risk that women receiving IPTp-SP are likely to experience infection with resistant parasites, we combine our burden estimates with estimates of the spatial distribution of SP resistance mutations in 2010 (the most up-to-date estimates currently available) [29] and estimates of how the parasitological efficacy of SP is affected by these mutations [22]. We then compare the impact of IPTp-SP at current coverage levels and if the intervention was scaled up to match ANC coverage levels observed in population-based health surveys.

## Methods

### Pregnancies at risk and potential burden by level of resistance

Published estimates of changes in the prevalence of malaria in children aged 2–10 y between 2000 and 2015 (estimated using a spatiotemporal geostatistical model fitted to geolocated prevalence data from 27,573 population clusters between 1994 and 2014) were obtained from the Malaria Atlas Project (MAP) at a 1 km × 1 km spatial resolution [5].

These estimates, along with fertility patterns calculated from population-based cross-sectional surveys such as Demographic and Health Surveys and Multiple Indicator Cluster Surveys [30] and estimates of the population of women of childbearing age in 2015 (based on LandScan 2007 population estimates [31], adjusted for population growth using UN projections and multiplied by UN estimates for the proportion of women of childbearing age [32]), were used to parameterise a previously published model of the relationship between malaria transmission within the population and exposure to malaria, risk of placental infection with *P*. *falciparum*, and mLBW during pregnancy [13,33] (see S1 Appendix for full details of this model). To estimate the extent to which current interventions are still required to protect pregnant women from malaria, this model was then used to estimate and map changes in the intrinsic risk and LBW burden of malaria (that which would exist in the absence of pregnancy-specific interventions) that have occurred in Africa between 2000 and 2015.

Molecular data compiled by the Worldwide Antimalarial Resistance Network have been used to generate maps showing the estimated prevalence of the quintuple K540E (based on 238 surveys) and the sextuple A581G (based on 124 surveys) SP resistance mutations within infected people across Africa [29]. We used these estimates and the above maps of MiP burden in order to provide a visual indication of where areas of high intrinsic MiP burden coincide with areas of high SP resistance. We then multiplied these spatial estimates of risk of malaria infection and mLBW by the prevalence of the two resistance markers (*dhps*-K540E and *dhps*-A581G) to estimate the number of pregnancies infected with highly SP resistant genotypes and how these correspond to the distribution of intrinsic mLBW risk.

We also estimated the intrinsic risk and burden according to categorisations based upon the observed changes in in vivo parasitological SP efficacy as defined by Desai et al. [22]. These original categorisations were based upon the frequency (the proportion of resistant parasites within the parasite population) of the quintuple resistance allele, whereas the spatial distribution of the mutation in [29] is estimated based upon prevalence (the proportion of infections containing resistant parasites). The relationship between these measures is unlikely to be straightforward. As a result, in this analysis we use the following definitions of the relationship between the frequency-based categorisation used by Desai et al. [22] and categories based upon prevalence of resistance. In these definitions, we make an attempt to keep our results conservative with respect to the effectiveness of IPTp-SP in the presence of resistance. Low quintuple mutation is defined as a frequency of the K540E allele less than 50% and a prevalence of K540E less than 15%. Intermediate quintuple mutation is defined as a frequency of the K540E allele between 50% and 90% and a prevalence of K540E between 15% and 80%. High quintuple mutation is defined as a frequency of the K540E allele greater than 90% and prevalence of K540E greater than 80%. In light of concerns about the effect of the sextuple *dhps*-A581G mutation upon SP efficacy, and in the absence of in vivo efficacy data in areas where this mutation is most prevalent, we added a further category: established sextuple mutation, where a prevalence of K540E above 80% coincides with a prevalence of A581G in excess of 10%.

In our estimates of MiP and LBW risk, we incorporate our uncertainty with respect to the relationship between transmission and these risks, which takes into account uncertainty in the risk of peripheral infection, the risk and chronicity of placental infection, and subsequent mLBW, and uncertainty in the MAP estimates of underlying prevalence. Although computational constraints did not allow us to calculate the precise joint posterior of these estimates, in an attempt to be conservative with respect to the uncertainty contained within our estimates, we here report the interval between (1) the lower bound 95% credible interval (crI) of our model of pregnancy using the lowest overall prevalence estimates for Africa on the basis of 100 realisations of these estimates from the joint posterior distribution of MAP estimates [5] and (2) the higher bound based upon the highest overall prevalence of these realisations. The absence of such realisations for the prevalence of resistance mutations prevents us from incorporating uncertainty in these estimates, and this limitation, along with others concerning these data, is returned to in the Discussion. To reflect our uncertainty regarding where the sextuple mutation has become established, we also report pregnancies at risk and mLBW burden in areas where prevalence of this mutation was estimated to be above 5% in 2010.

### Estimating the effectiveness of IPTp-SP given sulphadoxine-pyrimethamine resistance

We used a maximum-likelihood-based estimate of the efficacy of IPTp-SP against mLBW when the parasite remains fully sensitive to SP. We jointly fitted (1) the proportion of LBW that is mLBW and (2) the efficacy of IPTp-SP against mLBW (in the absence of either quintuple or sextuple resistance haplotypes). This efficacy estimate was fitted to all-cause LBW data from five randomised clinical trials (in order to best reflect IPTp-SP efficacy prior to any resistance driven by its implementation, we deliberately restrict our analysis to trials occurring prior to the intervention being adopted in the country where the trial took place [34]) where women received two doses of IPTp-SP [35–39]. The current recommendation is to provide the drug at every scheduled ANC visit from the second trimester onwards, and additional doses show an incremental impact on LBW [9], meaning our estimates of IPTp-SP efficacy are likely to be conservative with respect to this factor in settings with regular ANC attendance and SP provision. These estimates take into account that a proportion of LBW in these trials would have been caused by factors other than malaria, and therefore would not have been preventable even with an entirely efficacious antimalarial intervention (see S2 Appendix for full details of our estimates of IPTp-SP efficacy).

Estimates obtained from this fitting suggest that either (1) the malaria-attributable fraction of LBW of 8%–20% estimated in areas of stable malaria transmission is correct [2] (but if so, this implies that two-dose IPTp-SP has near-perfect efficacy against mLBW) or (2) malaria was the cause of a higher population attributable fraction (PAF) of LBW than the 8%–20% that has previously been estimated [2] (which allows less than perfect IPTp-SP efficacy). The first possibility is contradicted by the observed increase in efficacy of more frequently dosed regimens [9], and thus we derived a baseline estimate of 73.5% efficacy against mLBW, reflecting the assumption that 40% of LBW is attributable to malaria in these trials. We then varied the efficacy of two-dose IPTp-SP against mLBW between values of 48.4% and 97.5%, reflecting our remaining uncertainty in both the efficacy of the intervention against mLBW and the proportion of LBW that is mLBW in the trial settings (this interval includes the most likely estimate of efficacy for PAF values ranging between 30% and 60%). For comparison, trials of the impact of ITNs in similar settings [40,41] have shown an efficacy of 20% [42] against all-cause LBW, which translates to a 50% reduction in mLBW if malaria is responsible for 40% of LBW (see S1 Appendix for a full discussion of the results of this analysis and justification of parameters used).

In order to model the impact of resistance upon the ability of SP to clear existing infections among women receiving IPTp-SP, we used data on the impact of the differing levels of resistance defined by Desai et al. [22] to estimate the probability of recrudescence and the duration of prophylaxis following treatment with SP among asymptomatic parasitaemic women. In the absence of data as to how parasitological efficacy relates to the efficacy of IPTp-SP against mLBW, we made the assumption that any day of placental infection prevented contributes equally to LBW efficacy, regardless of factors such as gestational age or duration of infection. We denote the efficacy of IPTp-SP against LBW when the parasite remains fully sensitive as ε_*s*_ and the reduction in average time spent infected due to the intervention as $\delta_{s}=1-\frac{t_{s}}{t_{0}}$, where *t*_0_ is the average duration of infection in the absence of intervention and *t*_*s*_ is the average duration of infection when IPTp-SP is administered to fully sensitive parasites. Under this assumption, the efficacy of IPTp-SP against mLBW in the presence of resistance is then $\varepsilon_{r}=\varepsilon_{s}\frac{\delta_{r}}{\delta_{s}}$, where $\delta_{r}=1-\frac{t_{r}}{t_{0}}$, with *t*_*r*_ the average duration spent infected in the presence of resistance.

Due to the lack of data from settings where the sextuple mutation has also become established, and to be conservative with respect to the further need for interventions to protect women from MiP in addition to IPTp-SP in these settings, we did not include any LBW births averted within these settings within our final calculations. We then quantified our uncertainty in these areas by making two assumptions and reporting estimates obtained with both. At one extreme, we assumed that IPTp-SP has zero efficacy in these settings and, at the other, that IPTp-SP has the same efficacy in settings with established sextuple mutation as in settings with high prevalence of the quintuple mutation but where the sextuple mutation has not become established.

### Calculating the incremental impact of scale-up of IPTp-SP to match antenatal care coverage

Current IPTp-SP and ANC coverage were calculated across sub-Saharan Africa, where possible at a sub-national level, using data from the most recent available population-based surveys since 2010 such as Demographic and Health Surveys, Malaria Indicator Surveys, and Multiple Indicator Cluster Surveys (see S2 Appendix for full details). To be consistent with our estimates of efficacy, we calculated the proportion of women receiving IPTp-SP as the proportion receiving at least two doses of SP from the second trimester onwards. The incremental value of enhancing IPTp-SP uptake in the absence of other MiP-specific interventions (including using ITNs) was then calculated as the per pregnancy effectiveness against mLBW of IPTp-SP, multiplied by the number of women with at least three ANC visits who did not receive IPTp-SP.

For 26 countries in which individual-level data on ITN use were available, we calculated ITN usage by parity before and during pregnancy to estimate the proportion of potential mLBW deliveries that are currently being reached by ITNs across these surveys. We stratified by urban and rural in datasets containing this information. We then applied an estimate of ITN efficacy derived from trial data [40,41] in order to assess the extent to which ITNs are likely to be already preventing the potential LBW deliveries that would be reached by scaling up IPTp-SP coverage to all women with at least three ANC visits (see S2 Appendix for full details).

Our model incorporates the development of immunity over successive infections during pregnancy, the acquisition of which can be altered due to interventions. However, for ease of interpretation and in view of the historically low coverage of IPTp-SP and the difficulties associated with calculating burden when intervention in one pregnancy leads to higher risk in another, we viewed capturing these effects as beyond the scope of this analysis.

## Results

### Changing risk of malaria in pregnancy and associated low birthweight between 2000 and 2015

**Fig 1:**
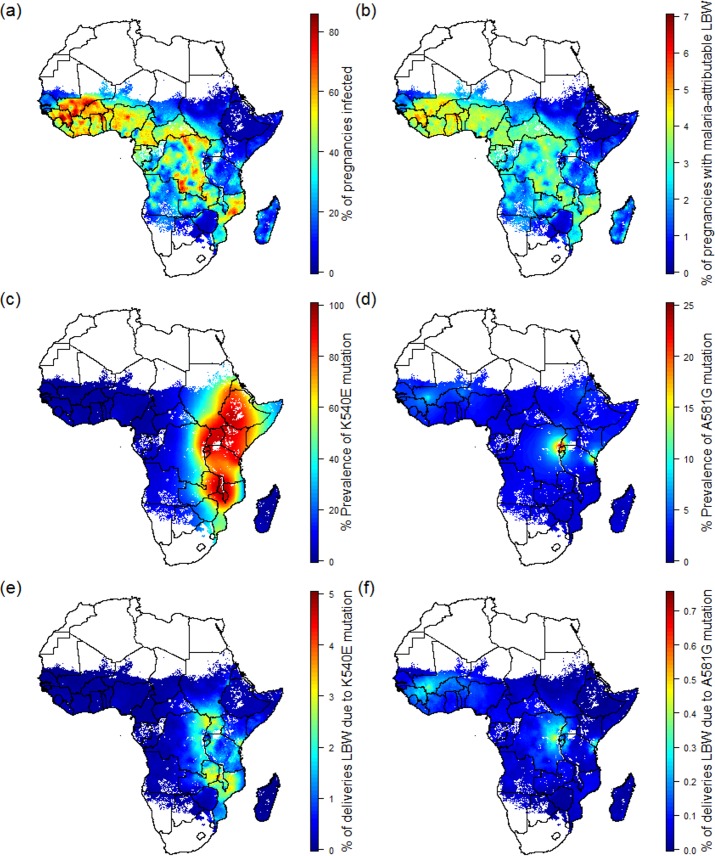
Preventable malaria burden and sulphadoxine-pyrimethamine resistance. (A) Percent of pregnancies infected in the absence of intervention in 2015. (B) Percent of pregnancies leading to malaria-attributable LBW in 2015 in the absence of intervention. (C) Prevalence of the quintuple K540E mutation within infected individuals. (D) Prevalence of the sextuple A581G mutation within infected individuals. (E) Percent of pregnancies leading to malaria-attributable LBW due to infection involving the quintuple mutation. (F) Percent of pregnancies leading to malaria-attributable LBW due to infection involving the sextuple mutation. LBW, low birthweight.

Taking into account population growth and the most recent estimates of fertility patterns across Africa, 30.6 million pregnancies leading to live birth would have occurred in malaria-endemic areas in 2015. If transmission in 2015 was still at the levels seen in 2000, 15.1 million (14.3 million–16.1 million 95% crI) of these pregnancies would have been infected with malaria in the absence of protection (Fig 1A shows the spatial distribution of this risk), leading to 1.08 million (650,000–1.51 million 95% crI) additional LBW deliveries (Fig 1B). Taking into account changes in transmission that have occurred since then, our actual estimates for 2015 of this intrinsic risk in the absence of protection are 9.5 million (8.3 million–10.4 million 95% crI) pregnancies infected and 750,000 (390,000–1.12 million 95% crI) LBW deliveries. These estimates represent a 37.0% (33.2%–40.6% 95% crI) reduction in the average risk of being infected and a 30.6% (27.3%–34.2% 95% crI) reduction in the risk of having a mLBW delivery over the 15-y period. The lower proportional reduction in LBW burden is attributable to reduced levels of parity-dependent immunity to placental infection (as a consequence of the lower levels of transmission), which increases the risk of LBW for each infected pregnancy.

### Pregnancies at risk in areas of sulphadoxine-pyrimethamine resistance

As of 2010, the quadruple *dhfr*-51,59,108/*dhps*-A437G haplotype was widespread throughout sub-Saharan Africa [29], but the quintuple and sextuple haplotypes were found only in specific regions of Africa [29]. Whilst the quintuple haplotype had reached saturation in much of east and southern Africa (Fig 1C), it was relatively rare in west Africa and parts of central Africa. Estimates of the prevalence of the *dhps*-A581G mutation (a proxy for the sextuple haplotype in areas with near saturation of the quintuple haplotype) suggest that the sextuple haplotype had become established only in two main areas: one focused around much of Rwanda and Burundi, along with areas of bordering countries (Democratic Republic of the Congo, southwestern Uganda, and northwestern Tanzania), the other focused around the southeastern Kenyan and northeastern Tanzanian border (Fig 1D).

Estimates of the spatial distribution of these resistance mutations in 2010 do not appear to be positively correlated with *P*. *falciparum* malaria transmission in 2015 as estimated by MAP. In many of the regions of highest transmission in Africa, including west Africa, northern Angola, and Madagascar, the prevalence of the quintuple mutation is estimated to be very low (Fig 1E). In total, we estimate that 53.4% (16.3 million) of the 30.6 million pregnancies leading to live birth at risk of malaria in Africa in 2010 occurred in areas with low prevalence (<15%) of the quintuple mutation and where the efficacy of IPTp-SP is likely to be high. These settings represent approximately two-thirds of the total number of women (6.6 million [5.5 million–7.4 million 95% crI] of 9.5 million [8.3 million–10.4 million 95% crI]) likely to experience infection during pregnancy and around two-thirds of the burden of mLBW (0.51 million [260,000–0.76 million 95% crI] of 0.75 million deliveries [390,000–1.12 million 95% crI]) in the absence of protection (Table 1).

**Table 1:** Risk and burden of infection in 2015 by level of resistance.

Variable	Total in Sub-Saharan Africa	Resistance Category			
		Low Quintuple Mutation	Intermediate Quintuple Mutation	High Quintuple Mutation	Established Sextuple Mutation
WOCBA at risk (2010) (millions)	235.0	121.1 (51.5%)	55.9 (23.8%)	47.4 (20.2%)	10.6 (4.5%)
Pregnancies leading to live birth at risk (millions)	30.6	16.3 (53.4%)	7.4 (24.1%)	5.6 (18.1%)	1.4 (4.4%)
Primigravidae (millions)	6.2	3.2 (51.8%)	1.5 (23.6%)	1.3 (20.0%)	0.3 (4.4%)
Infected pregnancies in absence of intervention (millions)	9.5 [8.3–10.4]	6.6 [5.6–7.3] (69.3%)	1.6 [1.5–1.7] (17.4%)	1.0 [0.9–1.1] (10.6%)	0.3 [0.3–0.4] (2.7%)
Malaria-attributable LBW if no intervention (thousands)^¥^	750 [394–1,120]	511 [258–758] (68.3%)	127 [69–196] (17.0%)	89 [53–128] (11.8%)	23 [14–38] (3.1%)
Percent pregnancies receiving IPTp-SP	21.6%	25.3%	15.4%	21.0%	12.4%
Percent pregnancies ≥3 ANC visits	65.3%	69.1%	57.4%	61.8%	77.8%
Percent pregnancies ≥1 ANC visit	79.4%	81.1%	72.3%	79.1%	98.2%
Potential LBW births avertable with 100% IPTp-SP coverage (thousands)^†^	502 [285–730]	375 [192–545] (74.7%)	84 [61–121] (16.6%)	44 [32–63] (8.7%)	ND [0–13]^‡^
Potential LBW births averted with current coverage (thousands)^†^	128 [75–184]	98 [56–143] (76.1%)	17 [12–24] (13.3%)	14 [9–19] (10.5%)	ND [0–2]^‡^
Further potential LBW births averted by extending coverage to all ANC3 (thousands)^†^	215 [128–318]	160 [94–236] (74.2%)	37 [22–55] (17.2%)	19 [12–27] (8.6%)	ND [0–9]^‡^
Total potential LBW births averted by extending coverage to all ANC3 (thousands)^†^	344 [202–502]	257 [160–379] (74.9%)	54 [34–79] (15.7%)	32 [21–46] (9.4%)	ND [0–10]^‡^

Values in brackets are 95% credible intervals; values in parentheses are the proportion of the total in each resistance category. Low quintuple mutation: prevalence of K540E less than 15%. Intermediate quintuple mutation: prevalence of K540E between 15% and 80%. High quintuple mutation: prevalence of K540E greater than 80%. Established sextuple mutation: prevalence of K540E greater than 80% and prevalence of A581G greater than 10%.

^¥^Values in brackets are 95% credible intervals based on our model estimates of potential malaria-attributable LBW in the absence of intervention.

^†^Values in brackets are intervals based upon our estimates of uncertainty in the efficacy of IPTp-SP by resistance category and the posterior mean of potential malaria-attributable LBW in the absence of intervention.

^‡^Not calculated due to lack of data on IPTp-SP efficacy from such settings; values in brackets represent the range between point estimates under the assumption that IPTp-SP has zero efficacy and point estimates under the assumption that efficacy in areas with established sextuple mutation is the same as that in areas with high quintuple mutation.

ANC, antenatal care; ANC3, women attending antenatal care at least three times during pregnancy; IPTp-SP, intermittent preventive treatment of malaria in pregnancy with sulphadoxine-pyrimethamine; LBW, low birthweight; ND, not determined; WOCBA, women of childbearing age.

A further 7.4 million pregnancies, representing 24.1% of pregnancies and 17.0% of potential mLBW deliveries, were estimated to have occurred in settings with intermediate levels of the quintuple mutation (15%–80% prevalence of *dhps*-K540E). The remaining 6.9 million pregnancies occurred in areas of with high levels of the quintuple mutation, representing 13.3% of infected pregnancies and 14.7% of potential LBW deliveries. High prevalence of the quintuple mutation coincides with a high likelihood of being exposed to infection in settings such as southern Uganda and northeast Mozambique, but in much of Ethiopia, Kenya, and central Tanzania, the impact of any SP resistance is likely to be mitigated largely by the fact that few women are likely to be infected with malaria during pregnancy. With the exception of southwest Uganda, the two estimated main foci of sextuple resistance are not located in areas of particularly high transmission (Fig 1F), with the 1.4 million pregnancies (4.4% of the total population of pregnant women at risk) in settings where prevalence of the mutation was above 10% representing just 3.1% of the total potential mLBW deliveries in the absence of protection from MiP. The equivalent figures for prevalence of the mutation above 5% was 2.3 million pregnancies at risk (7.7% of the total), representing 6.1% of potential mLBW deliveries. For full details of these estimates stratified by resistance level, gravidity, region, and country, see S3 Appendix.

### Relationship between efficacy of IPTp-SP and malaria-attributable low birthweight at differing levels of resistance

**Fig 2:**
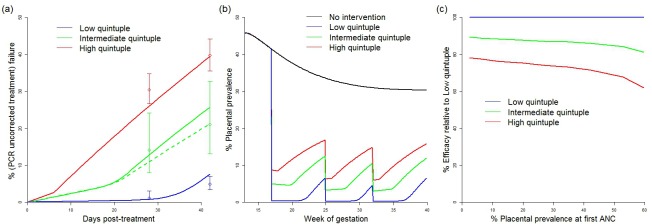
Impact of IPTp-SP upon low birthweight and effect of resistance. (A) Comparison between modelled (lines) and observed (circles with bars indicating 95% confidence intervals) 28- and 42-d failure rates in different resistance settings. Solid lines show a setting where the original prevalence at first ANC visit was 45% (replicating the high transmission settings in which these data were collected) [22]. The dashed green line shows a model simulation from a setting with 20% prevalence at first ANC visit (to reflect the relatively lower prevalence setting of Mansa, Zambia [22]). Prophylaxis in the model is assumed to last for an average duration of 28 d (low quintuple mutation), 14 d (intermediate quintuple mutation), and 7 d (high quintuple mutation). Recrudescent infections (estimated by the PCR-corrected failure rate, not shown) were assumed to reappear uniformly throughout the observation period. (B) Example simulations of placental prevalence by week of gestation in the absence of intervention, and where sulphadoxine-pyrimethamine is given at 17, 25, and 32 wk gestation at the different levels of resistance. (C) How efficacy of IPTp-SP against LBW is assumed to vary by resistance category and transmission intensity (based on changes in the estimated average time spent infected). ANC, antenatal care; IPTp-SP, intermittent preventive treatment of malaria in pregnancy with sulphadoxine-pyrimethamine; LBW, low birthweight.

The efficacy of IPTp-SP in preventing mLBW in areas of low quintuple mutation was estimated from trial data [35–39]. Treatment failure resulting in recrudescent infection by day 42 was incorporated in settings with low, intermediate, and high levels of the quintuple mutation (see Fig 2) With treatment failure at 1%, 9%, and 18%, respectively, the levels of reinfection observed were consistent with an average period of prophylaxis of around 28 d in settings with a low level of the mutation (consistent with observations of the prophylactic duration in children in such settings [43]) and around 7 d in settings where the K540E mutation has reached saturation. The prophylactic profile of 14 d used for treatment in areas of intermediate resistance is based on data from just one setting (Mansa, Zambia). However, as this setting has a K540E mutation frequency of 75% and is therefore likely to be towards the upper end of our definition of intermediate resistance, this seems likely to be a conservative estimate of the need for additional protection from reinfection following treatment in settings with intermediate resistance.

These estimates of the effect of resistance upon parasitological efficacy were then translated into a reduction in the overall efficacy of IPTp-SP against mLBW based upon the increase in the average exposure to infection within the model (see Fig 2C). With levels of transmission intensity reflective of the majority of settings, the reduction in efficacy was approximately 15% and 30% in areas of intermediate and high levels of quintuple mutation, respectively. These reductions in efficacy were then incorporated into our estimates of the impact of IPTp-SP scale-up in these settings.

### Insecticide-treated net coverage and the potential impact of scaling up IPTp-SP coverage in antenatal care

**Fig 3:**
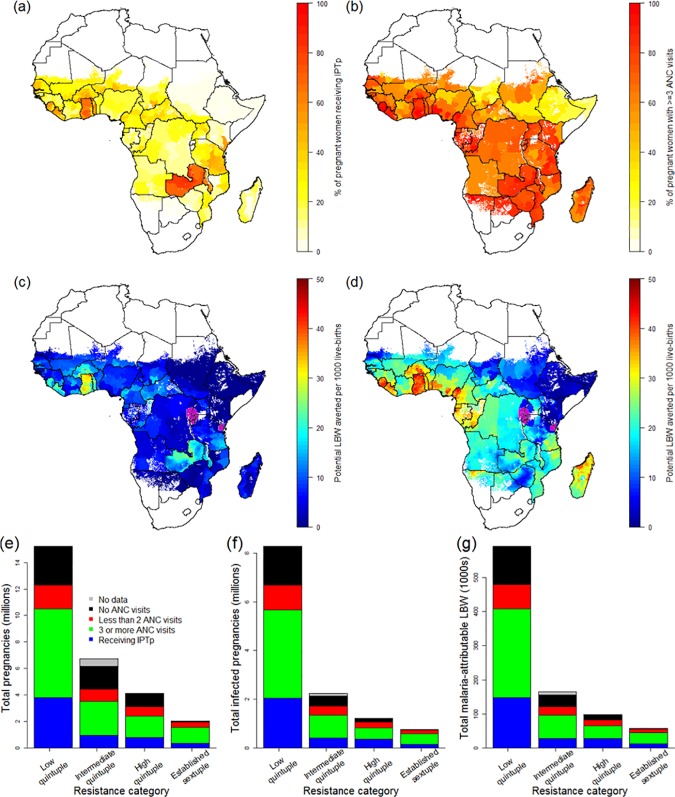
The current coverage of IPTp-SP and potential impact of scale-up. (A) Current coverage of IPTp (at least two doses of SP received at some stage during pregnancy) by first administrative unit according to the most recent population-based survey. (B) Current coverage of women visiting an ANC clinic at least three times during pregnancy. (C) Estimate of the current impact of IPTp-SP given current coverage, SP resistance, and transmission intensity (purple areas show settings where the sextuple mutation has become established, where we do not make an estimate of efficacy). (D) Estimate of the impact of IPTp-SP if given to all women visiting an ANC clinic at least three times, with purple areas as in (C). (E) Estimates of the total number of pregnancies receiving IPTp, and the ANC status of those who do not, by resistance category. (F) Equivalent estimates of total infected pregnancies. (G) Equivalent estimates of potential malaria-attributable LBW deliveries. ANC, antenatal care; IPTp, intermittent preventive treatment of malaria in pregnancy; IPTp-SP, intermittent preventive treatment of malaria in pregnancy with sulphadoxine-pyrimethamine; LBW, low birthweight; SP, sulphadoxine-pyrimethamine.

Combining estimates of the number of pregnancies at risk, coverage of IPTp-SP, and antenatal attendance at the first administrative level (Fig 3), we found that, according to the most recent population-based surveys, coverage of IPTp-SP remains very low: when weighted by population, across all areas of sustained transmission in mainland Africa, just 21.6% (15.2%–38.2% country-level interquartile range) of at risk pregnancies in 2015 received at least two courses of SP. There are only five countries where over 50% of pregnant women receive at least two courses of IPTp-SP during their pregnancy: Ghana, Liberia, Malawi, Togo, and Zambia (Fig 3A). In contrast, the proportion of women attending ANC clinics at least once during pregnancy is much higher (79.4% when weighted by population; 65.3% attend at least three times—Fig 3B). On this basis, the majority of women are likely to attend a clinic at least two times from the second trimester onwards and could therefore, in principle, receive IPTp-SP at least twice.

**Fig 4:**
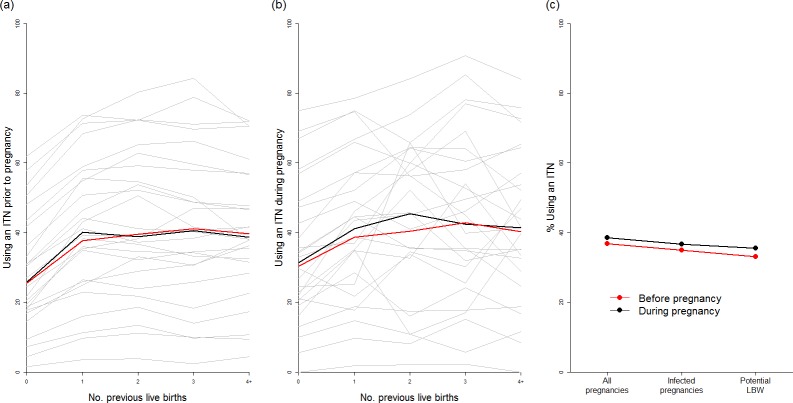
Usage of insecticide-treated nets in pregnancy. ITN usage prior to pregnancy (A) and during pregnancy (B) by parity (based on use during night preceding interview and weighted by age to reflect fertility patterns). Grey lines show usage estimates from each of the 26 country-level surveys with sufficient data, black lines show the median estimate of usage across surveys, and red lines show the estimate weighted by country population size, incorporating pixel-level urban and rural patterns. (C) Estimate of ITN usage weighted by population size within all pregnancies, within potentially infected pregnancies, and within pregnancies potentially leading to LBW. ITN, insecticide-treated net; LBW, low birthweight.

Our analysis of 26 population-based surveys of the use of ITNs before and during pregnancy demonstrates that, although ITN use was highly variable between countries (Fig 4A and 4B), the use of ITNs by women before and during their first pregnancy is generally substantially lower than use by women of higher parities. We found that the median of the survey-level bed-net use estimates immediately prior to first pregnancy was just 25%, with median usage rising to above 40% prior to and during subsequent pregnancies (Fig 4A). When we weighted these estimates by population, transmission, and parity (Fig 4C), we estimated that for all pregnancies that could potentially lead to mLBW births in these 26 countries, only 33% and 35% of women were using a net immediately prior to and during pregnancy, respectively.

Although the pattern of IPTp-SP uptake being substantially lower than ANC coverage is generally consistent across Africa (Fig 3; Table 1), both measures are generally higher than average in areas where the quintuple mutation is low (25.3% of pregnant women receive IPTp; 69.1% of pregnant women visit an ANC clinic at least three times during pregnancy). In 2010, in these least resistant settings, we estimate that current uptake of IPTp-SP would have prevented 98,000 (56,000–143,000 95% CrI) LBW live births in the absence of protection from ITNs. Furthermore, we estimate that if two-dose IPTp-SP had been administered to all women attending ANC at least three times (thus accounting for the fact that the first visit may be in the first trimester, when SP is contraindicated), 257,000 (160,000–379,000 95% crI) mLBW births would have been averted (Table 1) in these least resistant settings, representing an additional 160,000 (94,000–236,000 95% crI) LBW births averted compared to current levels of coverage. Taking into account the impact of resistance upon the efficacy of IPTp-SP against mLBW, we estimate a further 37,000 (22,000–-55,000 95% crI) and 19,000 (12,000–27,000 95% crI) additional LBW births averted in areas of intermediate and high levels of quintuple resistance, respectively (relative to a total of 42,000 [29,000–54,000 95% crI] and 28,000 [20,000–35,000 95% crI], respectively, if the parasite remained fully sensitive to the drug). This represents a reduction of 215,000 (128,000–318,000 95% crI) potential LBW deliveries across all resistance strata if IPTp-SP had been given to all women attending at least three ANC visits during pregnancy (see Table 1).

We found that, as there was little correlation between net use and IPTp-SP or ANC coverage in the 26 countries where sufficient data were available, only 33% of the estimated potential additional LBW births prevented through scaling up IPTp-SP would already have been covered by an ITN. Using the 50% estimate of ITN efficacy against mLBW, on the basis of a 40% PAF of LBW attributable to malaria in the two trials of ITNs during pregnancy, we estimate that just 16.5% of the additional mLBW births prevented by scaling up IPTp-SP in the absence of protection with ITNs in these countries are currently being prevented by ITNs, given the most recent estimates of coverage (see S1 Appendix for full details of this analysis).

## Discussion

Our estimates of the changing risk and burden of MiP across malaria-endemic sub-Saharan Africa support the notion that the observed declines in transmission in the 21st century coincided with reductions in the likelihood that women experienced malaria infection in pregnancy. However, our previous modelling suggested that, as transmission falls, among those who become infected, the average severity of MiP, and the risk of LBW, is likely to increase due to lower levels of immunity to placental infection [44]. This effect has recently been observed in data [45], and we estimate that proportional reductions in the burden of adverse outcomes due to MiP in terms of mLBW are likely to lag behind proportional reductions in the prevalence of infection in pregnancy [5]. As a result, MiP remains a substantial public health issue to address within most transmission settings and—because parity-dependent immunity means the first experience of MiP is substantially more severe than subsequent experiences—is likely to remain so until transmission is sufficiently low to ensure that women can live through their entire reproductive life without experiencing malaria during pregnancy at all.

The provision of interventions to prevent this burden remains inadequate. Our updated estimates of current IPTp-SP coverage suggest it remains low in most areas of sustained transmission [14]. This low coverage persists despite the high ANC coverage across much of Africa, including in areas where the quintuple mutation was not present. These settings, where SP efficacy remains near perfect, represent two-thirds of the intrinsic malaria burden to be averted. Combining these factors, we found that there were 7.1 million women in 2015 (23% of all pregnancies in sub-Saharan Africa) who did not receive ≥2 doses of IPTp-SP in areas where SP retains near perfect efficacy despite visiting ANC clinics at least three times during their pregnancy, representing 160,000 (94,000–236,000 95% crI) potential LBW deliveries that could have been prevented in a highly cost-effective manner [12] by ensuring regular administration of SP at ANC visits. Providing the intervention to these women should clearly be a public health priority. Although the quintuple resistance mutation (including *dhps*-K540E) substantially reduces the duration of prophylaxis provided by SP, it does appear that in the large majority of women infected with parasites with this mutation, SP retains sufficient effectiveness to clear infection, or at least substantially suppress parasite densities below detectable thresholds [22]. As a result, we estimate that even in settings where the quintuple mutation has reached saturation (but the sextuple has yet to become established), women are likely to substantially benefit from receiving SP. Consequently, our estimates strongly suggest that any successful increase in IPTp-SP coverage as a result of the Global Call to Action would be likely to lead to substantial (215,000 [128,000–318,000 95% crI] LBW deliveries averted in the absence of pregnancy-specific ITN use based on estimates of SP resistance in 2010) and cost-effective [12] improvements in newborn health in the large majority of malaria-endemic settings in Africa. This is the case even though we do not include the impact of IPTp-SP on other adverse maternal and birth outcomes due to malaria, such as preterm delivery, stillbirth, miscarriage, anaemia, and clinical malaria, which may also increase as transmission falls [7]. These efficacy data also suggest that our results would not be qualitatively different if the prevalence of the quintuple mutation was actually higher in 2010 (we do not incorporate uncertainty in this factor) or has increased since 2010 or is likely to increase with scale-up of the intervention (although previous analyses suggest that the areas with 80% prevalence of this mutation were actually declining between 2005 and 2010, when the intervention was first scaled up [29]).

When modelling the impact of IPTp-SP in areas of resistance, we made the assumption that the decrease in efficacy of IPTp-SP in preventing mLBW was proportional to the decrease in efficacy in preventing exposure to placental parasites throughout pregnancy (so efficacy against mLBW decreased approximately linearly with parasitological clearance rates in areas of lower transmission, with some additional decrease in efficacy against mLBW in areas of higher transmission due to the higher likelihood of reinfection throughout pregnancy—see S1–S3 Appendixes for more details). This assumption is based upon previous modelling suggesting that patterns of LBW observed in areas of sustained transmission in the absence of intervention are most reflective of the chronicity of infection within the placenta [44].

This assumption about the relationship between the changing parasitological efficacy of SP and the effectiveness of IPTp-SP against LBW is difficult to validate directly using data, however. On the one hand, observational studies show no declining trend in the effect of IPTp-SP upon LBW according to the level of K540E mutation, despite the declines in parasitological efficacy observed with increasing frequency of this mutation [9,22]; on the other hand, the only randomised clinical trial subsequent to scale-up in an area with appreciable quintuple resistance found no difference in LBW in those who received the intervention versus placebo despite IPTp-SP retaining substantial efficacy against peripheral and placental infection [46]. These results highlight the difficulty in attempting to interpret the relationship between such a highly malaria-specific endpoint, parasitological efficacy, and such a highly multi-factorial endpoint, all-cause LBW, which is likely to be influenced by various factors that are hard to control for, including the PAF of all-cause LBW due to malaria, the PAF of LBW due to other infections that are affected by any other antibiotic properties of SP, the overall distribution of birthweight within a setting, the degree and duration of immunity in multigravidae due to interventions in previous pregnancies, whether transmission has recently changed, and the potential effect of SP in suppressing parasite densities even if this does not lead to full parasite clearance. In the absence of data on the impact of these competing factors on the relationship between parasitological and non-parasitological endpoints, our analysis reflects what we believe is at least the most intuitive assumption about IPTp-SP efficacy (e.g., if IPTp-SP has 80% of its efficacy at preventing exposure to MiP, then at a population level, it will prevent approximately 80% of the LBW births it would prevent with no SP resistance).

These estimates also do not account for the burden of LBW that would have been averted in the absence of IPTp-SP, but we do not believe incorporating this factor would substantially alter our qualitative results for two reasons. First, the majority of the trials of IPTp-SP (upon which we based our estimates of IPTp-SP efficacy against mLBW) were in the context of women already using bed-nets, which may have made our estimates of the impact of IPTp-SP in the absence of ITNs conservative. Second, the current provision of ITNs to pregnant women also remains inadequate in most countries, with net usage by only 33% of all women whose pregnancies were estimated to otherwise lead to mLBW across the 26 countries in which sufficient data were available. Usage is particularly low in first-time mothers, those who are most at risk of severe adverse pregnancy outcomes if infected. These findings highlight the need to also improve access to and use of ITNs before and during pregnancy, and suggest that attempts to target LLINs to younger women, such as school-based distribution to adolescents, if successful at increasing uptake, would be highly effective at preventing subsequent morbidity in future years.

Finally, given the substantial estimated benefits of IPTp-SP, our point estimate that only 3% of pregnancies affected by malaria in 2015 occurred in areas where the sextuple A581G mutation had become established (>10% prevalence) by 2010 should not be cause for complacency. Previous studies have suggested that this mutation may be severely affecting the efficacy of SP [24,25,47]. If this is the case, with approximately double the number of women living in areas where prevalence had reached 5% by 2010, careful monitoring will be required to assess any spread of the mutation to the 25% of women living in areas where the quintuple mutation has already reached saturation (although a more recent review of the peer-reviewed literature published as of March 2016 has found that the A581G mutation has not been observed in any additional countries to those included our estimates [personal communication, Dr. Lucy Okell, Imperial College London, 18 January 2017]), along with studies allowing us to better understand the consequences of this mutation for IPTp-SP efficacy. Research into effective alternatives to IPTp-SP should also remain a high priority so that a contingency strategy is in place should further spread of the sextuple A581G mutation occur.

## Supporting information

S1 Appendix Estimating the impact of interventions. (DOCX)

S2 Appendix Modelling the risk and low birthweight burden of malaria in pregnancy. (DOCX)

S3 Appendix Estimates of impact by country, resistance category, and gravidity. (DOCX)

## Abbreviations

ANC: antenatal care
crI: credible interval
IPTp: intermittent preventive treatment of malaria in pregnancy
IPTp-SP: intermittent preventive treatment of malaria in pregnancy with sulphadoxine-pyrimethamine
ITN: insecticide-treated net
LBW: low birthweight
LLIN: long-lasting insecticidal net
MAP: Malaria Atlas Project
MiP: malaria in pregnancy
mLBW: malaria-attributable low birthweight
PAF: population attributable fraction
SP: sulphadoxine-pyrimethamine
