# Supplementary material for: Estimated impact on birth weight of scaling up intermittent preventive treatment of malaria in pregnancy given sulphadoxine-pyrimethamine resistance in Africa: A mathematical model
Source: PLoS Med. 2017 Feb 28;14(2):e1002243. doi: 10.1371/journal.pmed.1002243 (PMC5330448; doi:10.1371/journal.pmed.1002243)
Supplement: S1 Appendix — (DOCX) [file pmed.1002243.s001.docx]

**Estimated Impact on Birth Weight of Intermittent Preventive Treatment for Malaria in Pregnancy Given Sulphadoxine-Pyrimethamine Resistance in Africa: a Mathematical Model**

**Appendix 1 – Estimating the impact of interventions**

Full details of the model of the risk of malaria in pregnancy and malaria-attributable LBW have been published previously and are summarised in *Appendix 2*. In this appendix we provide a description of how we estimated IPTp-SP efficacy in the absence of SP resistance, how we calculated recent of IPTp uptake and ANC attendance across Africa. We then describe how we estimated ITN usage during and before pregnancy in setting where individual-level datasets were available and explore the relationship between ITN use and IPTp uptake.

**Estimating the efficacy of IPTp-SP in the absence of quintuple resistance**

In estimating the efficacy of IPTp-SP on low birth weight, it is also necessary to take into account the percentage of LBW that is attributable to malaria. The following describes a likelihood which can be fitted to trial results of the impact of IPTp-SP in order to estimate these quantities.

For the set of trials $T$ we define the number of deliveries and the number of which are LBW in the control arm of each trial as $N_{t}^{C}$ and $C_{t}$ respectively for each trial $t\epsilon T$ and $N_{t}^{Y}$ and $Y_{t}$ the equivalent values in the IPTp arm (see Table S1-1). We define $\theta_{t}$ as the prevalence of LBW in the absence of IPTp-SP and $\Theta$ as the set of these prevalences across all of the trials, with $\mu$ the population attributable risk of LBW due to malaria and $\varepsilon$ the efficacy of IPTp against malaria attributable LBW (mLBW).

The likelihood of the observed data across all of the trials is then

$$l\left( Y,C,N^{Y},N^{C}|\varepsilon,\mu,\Theta\right)=\prod_{t\epsilon T} {\theta_{t}}^{C_{t}}\left( 1-\theta_{t} \right)^{N_{t}^{C}-C_{t}}{q_{t}}^{Y_{t}}\left( 1-q_{t} \right)^{N_{t}^{Y}-Y_{t}}$$

where $q_{t}=\theta_{t}\left( \left( 1-\mu\right)+\mu(1-\varepsilon) \right)$.

The log of this likelihood was then maximised using the ‘mle’ maximum likelihood estimation function from the *stats4* package in R, which calculates an approximate covariance matrix by inverting the Hessian matrix at the optimum and thus finds the set of parameters that minimises the negative log-likelihood. Using this algorithm we found values of $\theta_{t}\epsilon\Theta$ the maximised the log-likelihood for all possible pairs of values of ε and μ between 0 and 100% in 0.1% increments (Figure S1-1).

**Table S1-1|**Data from trials used to estimate the PAR of LBW due to malaria and efficacy of IPTp against mLBW

| **First author** | **Year of Study** | **Setting** | **Control** | **Regimen** | **LBW in control arm** | | | **LBW in treatment arm** | | |
| --- | --- | --- | --- | --- | --- | --- | --- | --- | --- | --- |
|  |  |  |  |  | **Cases** | **Total** | **%** | **Cases** | **Total** | **%** |
| Schultz [1] | 1993 | Malawi | CQ | 2 dose IPT | 10 | 38 | 26.4 | 12 | 71 | 16.9 |
| Parise [2] | 1994-6 | Kenya | CM | 2 dose IPT | 52 | 340 | 15.3 | 27 | 325 | 8.3 |
| Njagi† [3] | 1997-9 | Kenya | Placebo | 2 dose IPT | 29 | 170 | 17.1 | 21 | 176 | 11.9 |
| Challis [4] | 2001-2 | Mozambique | Placebo | 2 dose IPT | 27 | 203 | 13.3 | 19 | 200 | 9.5 |
| Kayentao | 1998-01 | Mali | CQ | 2 dose IPT | 113 | 365 | 31.0 | 86 | 354 | 24.3 |

CQ= daily Chloroquine prophylaxis, CM=Case Management

†=Only women randomised to not receive ITNs included

When fitting our likelihood to the LBW data from five randomised control trials of IPT-SP, chosen on the basis that they were conducted prior to scale-up within each country in which the trial was based, we found that our two parameters of interest - the proportion of LBW attributable to malaria and the efficacy of IPTp-SP upon malaria-attributable LBW in these trials - were not identifiable from one another with pairs of negatively correlated values providing similarly good fits to the data (Figure S1-1). However, the fitted likelihood demonstrates that, when we discount LBW that would have occurred in the absence of malaria in these trials, either IPT is very effective at preventing LBW caused by malaria or malaria is responsible for a higher proportion of LBW in these malaria endemic settings than has typically been considered plausible.

For example, the population attributable risk (PAR) of LBW to malaria in areas of stable transmission has previously been estimated to be between 8 and 20% [5,6]. However in these trial data a PAR of 20% would be outside the 95% likelihood-based confidence interval even if we assume IPTp has 100% efficacy against LBW (Figure S1-1), Such a high level of efficacy seems implausible given that providing IPTp with a higher number of doses than those implemented in these trials has demonstrated incremental efficacy against LBW [7].

As a result, in this analysis we make assume a point estimate and uncertainty interval of IPTp efficacy against mLBW of 73.5% (48.4%-97.5%). This is equivalent to the maximum likelihood estimate and 95% confidence interval under the assumption that the PAR of malaria to LBW was 40% within the trial settings and also encompasses the maximum likelihood estimate of IPT efficacy upon mLBW for PARs between 30% and 60%.

Using similar reasoning, we also estimated the efficacy of ITN on mLBW using data from trials conducted in areas of very similar malaria endemicity [3,8], which demonstrated a reduction in all cause LBW of 20% (0-34% C.I.) relative to a control of no net [9]. Assuming that 40% of LBW is attributable to malaria, this suggests an estimate of ITN efficacy against mLBW of 50% (0-85%).


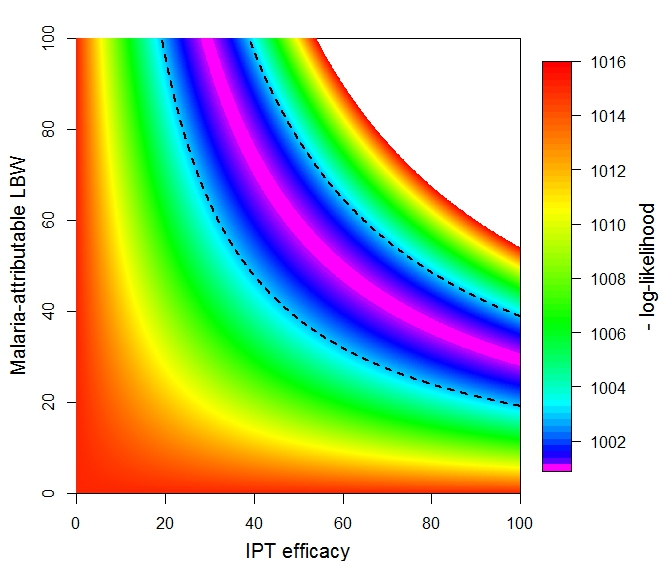


**Figure S1-1|Estimating the efficacy of IPTp-SP against mLBW in absence of quintuple mutation**. Figure shows the negative log-likelihood of models assuming different proportions of malaria-attributable LBW and associated efficacies of IPT-SP against this proportion. The purple line shows the best fitting pairs, dashed lines show 95% confidence intervals around these

**Calculating current coverage of IPTp and Antenatal care**

Data on the current coverage of IPTp and ANC were obtained from the most recent population-based health survey (DHS, MIS or MICS) since 2009. These are highly standardised and widely used estimates, including by WHO when compiling the World Malaria Report [10] and previous analyses of IPTp coverage [11,12], used to measure a wide range of health indicators including those relevant to malaria. In the cases of measures of ANC and IPTp coverage women in randomly selected clusters are interviewed and asked, if having reported having a pregnancy in the two years preceding the interview, about the ANC care they received, whether they received SP and how many times they were provided the drug. There surveys were powered to provided sub-national estimates on IPTp uptake for 36 of 43 countries with endemic malaria transmission in mainland Africa and Madagascar and the proportion of women attending ANC at least 3 times for 37 of them (see Table S1-1). For ANC coverage, population-based health survey data on ANC attendance from 2008 was used for two further countries (Angola and Botswana). In some countries (Equatorial Guinea and Kenya estimates were only available for one or more ANC visits and four or more ANC visits. For these countries, estimates of 3 or more visits were interpolated from the best fitting linear relationship from all other subnational regions (see Figure S1-2).

**
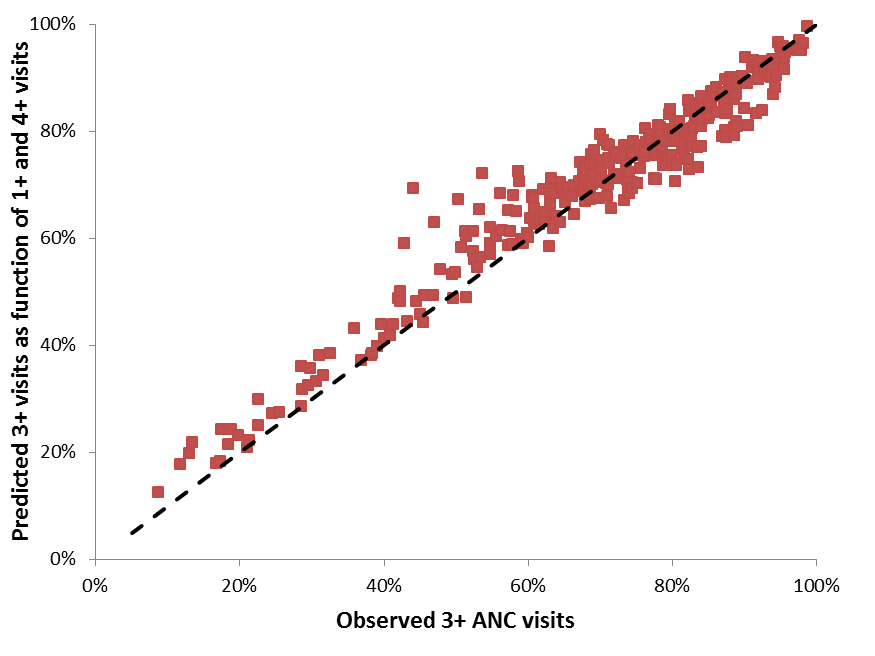
**

**Figure S1-2|Observed percentage of women visiting ANC at least three times during pregnancy compared to that predicted by best fitting linear combination of percentage visiting at least once and percentage visiting at least four times (ANC3plus=0.61*(ANC1plus)+0.40*(ANC4plus)).**

For IPTp coverage, calculated as the proportion of women receiving at least two doses of SP, with at least one dose through an ANC visit, counties which had no data and had no official control policy to implement IPTp-SP within the country (or in the case of Rwanda has officially halted this policy) according to the review by *van Eijk et al.* [11] we assumed coverage of IPTp-SP was zero. Where only country-level estimates of either IPTp or ANC coverage were available we applied these uniformly across the country (see Table S1-2).

**Table S1-2|Data sources used for subnational ANC,IPTp and ITN coverage estimates**

| **Country** | **ANC data source** | **IPTp data source** | **ITN data used^$^** |
| --- | --- | --- | --- |
| Angola | * | MIS 2011 | Not Available |
| Benin | DHS 2011-12 | DHS 2011-12 | DHS 2011-12 |
| Botswana | ¥¤ | † | Not Available |
| Burkina Faso | DHS 2010 | DHS 2010 | DHS 2010 |
| Burundi | DHS 2010 | DHS 2010 | DHS 2010 |
| Cameroon | DHS 2011 | DHS 2011 | DHS 2011 |
| CAR | MICS 2010 | MICS 2010 | Not Available |
| Chad | MICS 2010 | MICS 2010 | Not Available |
| Cote D'Ivoire | DHS 2011-12 | DHS 2011-12 | DHS 2011-12 |
| DRC | DHS 2013-14 | DHS 2013-14 | DHS 2013-14 |
| Djibouti | § | † | Not Available |
| Equatorial Guinea | MIS 2011¤ | MIS 2011‡ | Not Available |
| Eritrea | § | † | Not Available |
| Ethiopia | DHS 2011 | † | Not Available |
| Gabon | DHS 2012 | DHS 2012 | DHS 2012 |
| Gambia | DHS 2013 | DHS 2013 | DHS 2013 |
| Ghana | MICS 2011 | MICS 2011 | Not Available |
| Guinea | DHS 2012 | DHS 2012 | DHS 2012 |
| Guinea-Bissau | MICS 2010 | MICS 2010 | Not Available |
| Kenya | DHS 2014‡¤ | DHS 2014‡ | Not Available |
| Liberia | DHS 2013 | DHS 2013 | DHS 2013 |
| Madagascar | MIS 2013 | MIS 2013 | MIS 2013 |
| Malawi | DHS 2010 | DHS 2010 | DHS 2010 |
| Mali | DHS 2012-3 | DHS 2012-3 | DHS 2012-3 |
| Mauritania | MICS 2011 | MICS 2011 | Not Available |
| Mozambique | DHS 2011 | DHS 2011 | DHS 2011 |
| Namibia | DHS 2013 | DHS 2013 | DHS 2013 |
| Niger | DHS 2012 | DHS 2012 | DHS 2012 |
| Nigeria | DHS 2013 | DHS 2013 | DHS 2013 |
| Republic of Congo | DHS 2011-12 | DHS 2011-12 | DHS 2011-12 |
| Rwanda | DHS 2010 | † | DHS 2010 |
| Senegal | DHS 2012-13 | DHS 2012-13 | DHS 2012-13 |
| Sierra Leone | DHS 2013 | DHS 2013 | DHS 2013 |
| Somalia | MICS 2011^£^ | MICS 2011^£^ | Not Available |
| South Africa | § | † | Not Available |
| Sudan | MICS 2010 | MICS 2010 | Not Available |
| S Sudan | MICS 2010 | MICS 2010 | Not Available |
| Swaziland | MICS 2010 | MICS 2010 | Not Available |
| Tanzania | AIS/MIS 2011-12 | AIS/MIS 2011-12 | DHS 2010 |
| Togo | DHS 2013-14 | DHS 2013-14 | DHS 2013-14 |
| Uganda | DHS 2011 | DHS 2011 | DHS 2011 |
| Zambia | DHS 2013-4 | DHS 2013-4 | DHS 2013-4 |
| Zimbabwe | DHS 2011 | DHS 2011 | DHS 2011 |

**^$^**Requires individual-level data to take into account parity patterns of ITN use

*Country-level estimates from the Integrated Survey on the Welfare of Population 2008-2009 [13]

¥Country-level estimates from the Botswana Family Health Survey 2007-2008 [14]

¤Proportion of pregnant women with at least 3 ANC visits estimated from fitted relationship with proportion visiting at least 1 and 4 ANC visits (see below).

†No available population-based survey estimates and listed as not having a policy of IPTp-SP in [12] so IPTp-SP coverage assumed to be zero.

^£^Estimates for Northeast Zone and Somaliland only, no data was found for the remainder of the country.

§No data source found

‡Only country-level estimates are available.

**Estimating the existing impact of ITNs upon mLBW and relationship to IPTp uptake**

We identified 26 African countries where we could gain access to individual-level data from a post-2009 DHS or MIS survey. In these surveys we then stratified use of an ITN in the previous night by age, pregnancy status, previous number of live-born babies, urban or rural status and, where possible, use of IPTp and access to antenatal care in a previous pregnancy (see Table S1-2).

Within each of the 26 surveys, the use of ITNs at the beginning of pregnancy was estimated by based on the use of nets in non-pregnant women, stratified according to the number of previous live-born babies, urban/rural status and five-year age interval. Parity and urban/rural specific estimates were then weighted according to the rates at which women in each stratum become pregnant.

Within the model, we then assign ITNs to women within each setting based on these these usage estimates according to the number of previous pregnancies they have had and whether they live within an urban or rural setting. If this woman is estimated to have a mLBW in the absence of intervention this is then averted with a probability equivalent to the efficacy of ITNs against LBW based upon trial data (see above) to provide an estimate of the proportion of potential mLBW occurring in 2010 which would have been prevented by ITNs given current levels of coverage in women of child-bearing age.

When estimating the effect ITNs are likely to have upon our estimates of the impact of IPTp scale-up we calculated 1) the number of women with three or more ANC visits not receiving IPTp in each country and multiplied this by 2) our estimate of the proportion of the total mLBW burden that occurs in women using a net immediately prior to pregnancy. This accounts for any correlation between ANC or IPT uptake and ITN use at the country-level. However, as these measures do not appear to be heavily correlated, this factor had little impact upon our estimates of effective coverage of ITNs in these women (Figure S1-2)).


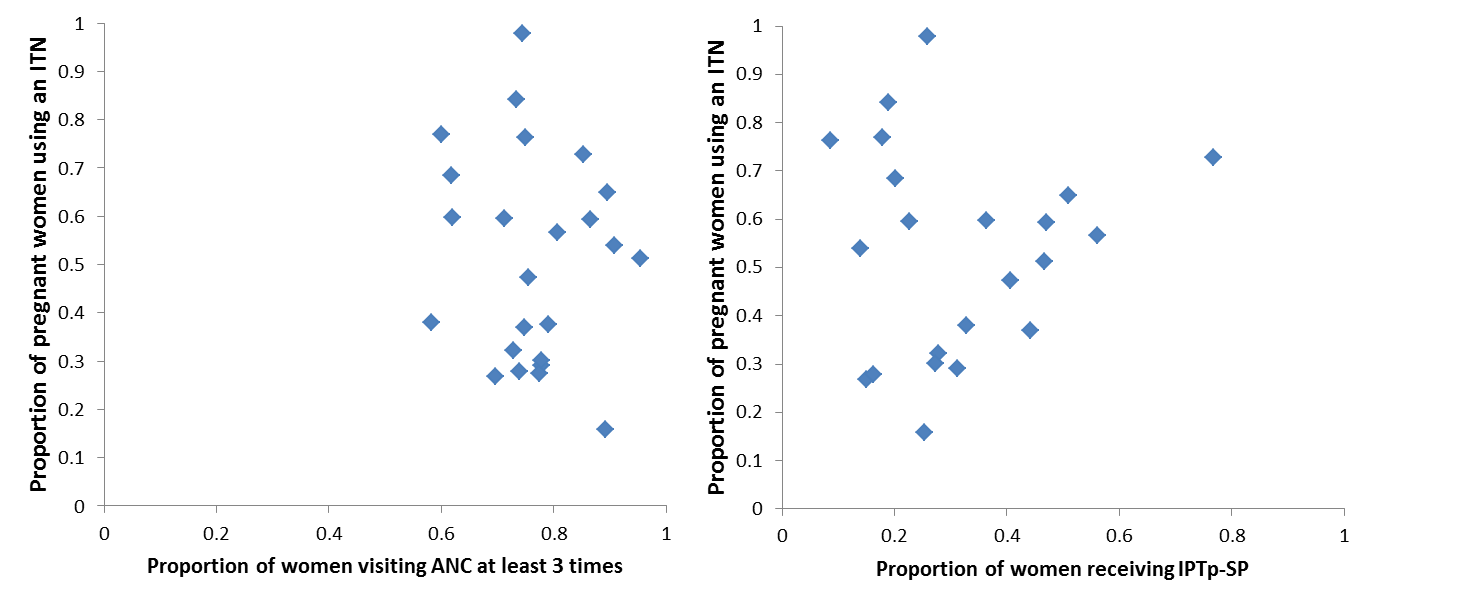


**Figure S1-2|Relationship between country-level ITN use in pregnancy and uptake of IPTp-SP.** Data from most recent country-level population-based survey (DHS or MIS).

**References**

1. Schultz LJ, Steketee RW, Macheso A, Kazembe P, Chitsulo L, Wirima JJ. The efficacy of antimalarial regimens containing sulfadoxine-pyrimethamine and/or chloroquine in preventing peripheral and placental Plasmodium falciparum infection among pregnant women in Malawi. Am J Trop Med Hyg. 1994;51: 515–22. Available: http://www.ncbi.nlm.nih.gov/pubmed/7985742

2. Parise ME, Ayisi JG, Nahlen BL, Schultz LJ, Roberts JM, Misore A, et al. Efficacy of sulfadoxine-pyrimethamine for prevention of placental malaria in an area of Kenya with a high prevalence of malaria and human immunodeficiency virus infection. Am J Trop Med Hyg. 1998;59: 813–22. Available: http://www.ncbi.nlm.nih.gov/pubmed/9840604

3. Njagi JK. The effects of sulfadoxine-pyrimethamine intermittent treatment and pyrethroid impregnated bed nets on malaria morbidity and birth weight in Bondo district, Kenya. Nairobi: University of Nairobi; Copenhagen: Danish Bilharziasis Laboratory. 2002.

4. Challis K, Osman NB, Cotiro M, Nordahl G, Dgedge M, Bergström S. Impact of a double dose of sulphadoxine-pyrimethamine to reduce prevalence of pregnancy malaria in southern Mozambique. Trop Med Int Health. 2004;9: 1066–73. doi:10.1111/j.1365-3156.2004.01307.x

5. Steketee RW, Nahlen BL, Parise ME, Menendez C. The burden of malaria in pregnancy in malaria-endemic areas. Am J Trop Med Hyg. ASTMH; 2001;64: 28. Available: http://www.ncbi.nlm.nih.gov/pubmed/11425175

6. Guyatt HL, Snow RW. Malaria in pregnancy as an indirect cause of infant mortality in sub-Saharan Africa. Trans R Soc Trop Med Hyg. 2001;95: 569–76. Available: http://www.ncbi.nlm.nih.gov/pubmed/11816423

7. Kayentao K, Garner P, van Eijk AM, Naidoo I, Roper C, Mulokozi A, et al. Intermittent preventive therapy for malaria during pregnancy using 2 vs 3 or more doses of sulfadoxine-pyrimethamine and risk of low birth weight in Africa: systematic review and meta-analysis. JAMA. 2013;309: 594–604. doi:10.1001/jama.2012.216231

8. Ter Kuile FO, Terlouw DJ, Phillips-Howard PA, Hawley WA, Friedman JF, Kariuki SK, et al. Reduction of malaria during pregnancy by permethrin-treated bed nets in an area of intense perennial malaria transmission in western Kenya. Am J Trop Med Hyg. 2003;68: 50–60. Available: http://www.ncbi.nlm.nih.gov/pubmed/12749486

9. Gamble C, Ekwaru PJ, Garner P, ter Kuile FO. Insecticide-treated nets for the prevention of malaria in pregnancy: a systematic review of randomised controlled trials. Rogerson SJ, editor. PLoS Med. Public Library of Science; 2007;4: e107. Available: http://dx.plos.org/10.1371/journal.pmed.0040107

10. World Health Organization. WHO | World Malaria Report 2015. World Health Organization; Available: http://www.who.int/malaria/publications/world-malaria-report-2015/report/en/

11. Van Eijk AM, Hill J, Larsen DA, Webster J, Steketee RW, Eisele TP, et al. Coverage of intermittent preventive treatment and insecticide-treated nets for the control of malaria during pregnancy in sub-Saharan Africa: a synthesis and meta-analysis of national survey data, 2009–11. Lancet Infect Dis. 2013; Available: http://www.sciencedirect.com/science/article/pii/S1473309913701993

12. Van Eijk AM, Hill J, Alegana VA, Kirui V, Gething PW, Ter Kuile FO, et al. Coverage of malaria protection in pregnant women in sub-Saharan Africa: a synthesis and analysis of national survey data. Lancet Infect Dis. Elsevier Science, The Lancet Pub. Group; 2011;11: 190–207. Available: http://www.pubmedcentral.nih.gov/articlerender.fcgi?artid=3119932&tool=pmcentrez&rendertype=abstract

13. Instituto Nacional de Estatistica (INE). Integrated Survey on the Welfare of Population| Vol.1 [Internet]. Luanda; 2011. Available: http://www.childinfo.org/files/Angola_IBEP_2008-09_FinalReport_Vol1_Eng.pdf

14. Central Statistics Office. Country-level estimates from the Botswana Family Health Survey 2007-2008. Gaborone, Botswana; 2009.
