# Supplementary material for: Estimated impact on birth weight of scaling up intermittent preventive treatment of malaria in pregnancy given sulphadoxine-pyrimethamine resistance in Africa: A mathematical model
Source: PLoS Med. 2017 Feb 28;14(2):e1002243. doi: 10.1371/journal.pmed.1002243 (PMC5330448; doi:10.1371/journal.pmed.1002243)
Supplement: S2 Appendix — (DOCX) [file pmed.1002243.s002.docx]

**Estimated Impact on Birth Weight of Intermittent Preventive Treatment for Malaria in Pregnancy Given Sulphadoxine-Pyrimethamine Resistance in Africa: a Mathematical Model**

**Appendix 2 – Modelling the risk and LBW burden of malaria in pregnancy**

The model of malaria in pregnancy, its link to transmission intensity and fertility and association to low birthweight (LBW) is described in full elsewhere [1,2], along with full details of the data used to fit the model and the methodology used for fitting the model parameters to this data. In this appendix we provide a succinct description of the model, with full details of parameter values used in the analysis and briefly describe the data and fitting process that was undertaken, including relevant references to where further detail of these preceding analyses can be found. We first provide an abridged description of an established mathematical model of P.falciparum transmission [3]used to generate patterns of exposure to ongoing infection at the beginning of pregnancy and the incidence of infection throughout gestation. We then describe how the progression of placental infection [1] and the risk of malaria-attributable LBW (mLBW) in the absence of intervention [4] are incorporated within the model.

**Simulating patterns of exposure to P.falciparum in the general population**

The patterns of P.falciparum infection in the general population within a setting are generated using a deterministic, compartmental version of the model of *P. falciparum* transmission developed by Griffin et al. [3]. In this model, members of the general population can transition through six different infectious states: susceptible (S), untreated clinical disease (D), treated clinical disease (T) and protected due to prophylaxis from prior treatment (P), asymptomatic patent (detectable by slide microscopy) infection (A), and sub-patent asymptomatic infection (U). These transitions occur according to the rates shown in Supplementary Figure S1, following a latent period of 12 days in the case of a new infection [5].

**Supplementary Figure S2-1| Flow diagram for human stages of the general population model (taken from Griffin et al.**[3]**)**

The model is further subdivided by age and to account for local-level heterogeneity in transmission intensity. Acquired immunity then acts in three ways: to reduce the probability of clinical disease due to a new infection $\phi$, to lower the force of infection acting upon an age- and transmission-specific class, and to reduce the duration of time in which individuals have sufficiently dense infection to be detected by slide microscopy $\frac{1}{r_{A}}$.

*Protection from clinical disease.* This a combination of protection acquired from maternal antibodies, $I_{CM}$ and that acquired by exposure to infection $I_{CA}$. The latter depends upon the age-dependent force of infection and a constant decay rate $1/d_{c}$ thus:

|  | $\frac{\partial I_{CA}}{\begin{aligned} \partial t \end{aligned}}+\frac{\partial I_{CA}}{\begin{aligned} \partial a \end{aligned}}=\Lambda\left( a,t \right)-\frac{I_{CA}}{d_{C}},$ | [S1] |
| --- | --- | --- |

which constitutes the mean rate of increase in protection from clinical disease as a function of the age-dependent force of infection $\Lambda\left( a,t \right)$. Maternal antibodies are assumed to confer immunity to clinical disease proportionally to the immunity to clinical disease acquired by young adult women within the same location $\left( I_{CA}\left( 20,t \right) \right)$ with this maternally-derived immunity decaying at a constant rate, $1/d_{M}$, following birth:

|  | $\frac{{\partial I}_{CM}}{\begin{aligned} \delta t \end{aligned}}+\frac{\partial I_{CM}}{\begin{aligned} \delta a \end{aligned}}=-\frac{I_{CM}}{d_{M}}, I_{CM}\left( 0,t \right)={P_{CM}I}_{CA}\left( 20,t \right),$ | [S2] |
| --- | --- | --- |

where $P_{CM}$ is the level of immunity maternally-acquired relative to the level of immunity in young adult women.

This relates to the probability of experiencing clinical disease following infection according to the following function

|  | $\phi\left( a \right)=\frac{1}{1+\left( I_{C}(a)/I_{C0} \right)^{\kappa_{C}}},$ | [S3] |
| --- | --- | --- |

where $I_{C}\left( a,t \right)=I_{CA}\left( a,t \right)+I_{CM}\left( a,t \right)$ and $\kappa_{C}$ and $I_{C0}$ are estimated parameters.

These immunity parameters were estimated by fitting to the age-stratified parasite prevalence and clinical incidence data from 56 settings in Africa using prior estimates for the EIR in each location as previously described [3].

*Infection-blocking immunity.* This is assumed to increase in proportion to the entomological inoculation rate within a strata, $EIR(a,t)$, raised to the fitted power parameter $\tau$. It is assumed to wane at a fixed rate ${1/d}_{b}$:

|  | $\frac{\partial I_{B}}{\begin{aligned} \partial t \end{aligned}}+\frac{\partial I_{B}}{\begin{aligned} \partial a \end{aligned}}={EIR(a,t)}^{\tau}-\frac{I_{B}}{d_{b}}.$ | [S4] |
| --- | --- | --- |

The age-dependent probability of infection then follows a second Hill function:

|  | $b\left( a \right)=b_{MIN}+\frac{b_{MAX}-b_{MIN}}{1+\left( I_{B}(a)/I_{B0} \right)^{\kappa_{b}}}$ | [S5] |
| --- | --- | --- |

where $b_{MIN}$,$b_{MAX}$, $I_{B0}$ and $\kappa_{b}$ are parameters to be estimated. This then results in the force of infection

|  | $\Lambda\left( a,t \right)=EIR(a,t)\varsigma b(a)$ | [S6] |
| --- | --- | --- |

where $\varsigma$ is a measure of the biting rate experienced by an individual relative to population-level mean. This within-population heterogeneity in biting is then modelled according to a Log-Normal distribution with mean 1 such that:

|  | $\log\left( \varsigma\right)\sim N\left( -\sigma^{2}/2,\sigma^{2} \right),$ | [S7] |
| --- | --- | --- |

where the variance $\sigma^{2}$ is estimated during the model fitting. For the compartmental version of the model used here this heterogeneity was approximated using a Gauss-Hermite approximation using 5 separate heterogeneity strata.

*Blood-stage immunity*. This reduces parasite density and hence the duration of patent infection. It is assumed to develop with age and decay at a constant rate $1/d_{A}$. This gives the following rate of progression from patent to sub-patent infection:

|  | $r_{A}\left( a \right)=r_{A0}\left( 1+(w_{A}-1)\frac{\left( I_{A}/I_{A0} \right)^{\kappa_{A}}}{1+\left( I_{A}/I_{A0} \right)^{\kappa_{A}}} \right),$ | [S8] |
| --- | --- | --- |

where $I_{A}$ is the level of parasite immunity and values for $r_{A0}$, $\kappa_{A}$, $I_{A0}$ and $w_{A}$ were obtained from previous model fitting to data from the Garki project [6].

*Mosquito model* The dynamics of infection within the mosquito vectors was modelled by partitioning the population into three stages, susceptible $S_{M}$, latently infected $E_{M}$ and infectious $I_{M}$ with the density relative to the human population in each category progressing according to the following equations:

|  | $\frac{dS_{M}}{dt}=-\Lambda_{M}S_{M}+B-\mu S_{M}$ $\frac{dE_{M}}{dt}=\Lambda_{M}S_{M}-\Lambda_{M}\left( t-\tau_{M} \right)S_{M}\left( t-\tau_{M} \right)P_{M}-\mu E_{M}$ $\frac{dI_{M}}{dt}=\Lambda_{M}\left( t-\tau_{M} \right)S_{M}\left( t-\tau_{M} \right)P_{M}-\mu I_{M}$ | [S9]  [S10]  [S11] |
| --- | --- | --- |

In this analysis we assume constant EIR in each setting, which we ensure by keeping the birth rate of susceptible mosquitoes constant (i.e. $B=\mu N_{M}$, with $N_{M}=S_{M}+E_{M}+I_{M}$). $\tau_{M}$ is the extrinsic incubation period with $P_{M}=\exp\left( - \mu\tau_{M} \right)$ representing the proportion of infected mosquitoes surviving to become infectious. The force of infection acting upon mosquitoes,$\Lambda_{M}$ is obtained by summing the infectivity to mosquitoes across all of the population with infection probabilities from feeding on a human in each infection stage obtained by fitting the transmission cycle to biting rate and parasite prevalence data.

To be fully reproducible the values of the parameters used in this model for this analysis are listed in Supplementary Table S2-1. We include references for values when they are taken directly from the literature. When they are obtained from previous model-fitting we report the posterior distribution and refer the reader to the original paper for full details of how these values were obtained (Note that in order to fit the model of the progression of placental infection and risk of LBW we only use the posterior median of these intervals).

**Supplementary Table S2-1 Model parameters of malaria transmission in the general population**

| Symbol | Description | Posterior Median (with 95% credible interval) | Reference |
| --- | --- | --- | --- |
| **Human infectious periods and infectivity to mosquitoes** | | | |
| $f_{t}$ | Fraction of cases treated | 60% | [7] |
| $1/r_{D}$ | Duration of disease without treatment | 5 days (Fixed) | [8] |
| $1/r_{T}$ | Duration of infectivity post treatment | 5 days (Fixed) | [9] |
| $1/r_{A}$ | Duration of patent infection | 200 days (Fixed) | [5,10] |
| $1/r_{U}$ | Duration of sub-patent infection | 107.9 (92.3-122.8) | [1] |
| $1/r_{P}$ | Duration of prophylaxis due to treatment of disease outside of pregnancy | 25 days | assumed |
| * | Infectivity to mosquitoes in state D | 0.40 per infectious bite | [3] |
| * | Infectivity to mosquitoes in state A | 0.12 per infectious bite | [3] |
| * | Infectivity to mosquitoes in state U | 0.02 per infectious bite | [3] |
| * | Infectivity to mosquitoes in state T | 0.12 per infectious bite | [3] |
| * | Delay from emergence of blood-stage parasites to onward infectivity | 12.5 days (Fixed) |  |
| **Parameters describing acquistion of immunity** | | | |
| $b_{MAX}$ | Probability of human infection from an infectious bite with no immunity | 0.93 (0.84-0,98) | [1] |
| $b_{MIN}$ | Probability of human infection from an infectious bite with full immunity | 0.006 (0.004,0.01) | [1] |
| $d_{b}$ | Decay parameter for infection blocking immunity | 10 years | [3] |
| $I_{B0}$ | Infection blocking immunity scale parameter | 1072.1 (916.3, 1231.5) | [1] |
| $\kappa_{b}$ | Infection blocking immunity shape parameter | 6.05 (5.17, 7.21) | [1] |
| $\tau$ | Rate of acquisition of Infection blocking immunity power scaling parameter | 0.21 (0.17-0.24) | [1] |
| $I_{C0}$ | Protection from clinical disease scale parameter | 53.03 (41.65,66.64) | [1] |
| $\kappa_{c}$ | Protection from clinical disease shape parameter | 2.01 (1.54,2.80) | [1] |
| $P_{CM}$ | Immunity level of newborn relative to mother | 0.53 (0.25,0.88) | [1] |
| $d_{M}$ | Decay parameter for maternal immunity | 230.4 (143.9,348.0) days | [1] |
| $1/r_{A0}$ | Duration of patent infection with maximum immunity | 160 days | [3] |
| $d_{A}$ | Decay parameter for blood-stage immunity | 10 years | [3] |
| $I_{B0}$ | Blood-stage immunity scale parameter | 4732.5 | [3] |
| $\kappa_{b}$ | Blood-stage immunity shape parameter | 5 | [3] |
| **Mosquito parameters** | | | |
| $\sigma^{2}$ | Variance of log of heterogeneity in biting rates | 1.04 (0.83-1.27) | [1] |
| $\tau_{M}$ | Extrinsic Incubation period | 10 days | [11] |
| * | Biting rate | 0.3333/day | [12] |
| $1/\mu$ | Mean life expectancy | 7.6 days | [13] |

*Parameter symbol not defined in abridged model description

**Generating patterns of exposure to P.falciparum in pregnant women**

Individual-level data for the age at which women most recently gave birth in the previous 36 months were obtained for the most recent population-based survey (e.g. DHS/MIS) in each country and transformed into age of beginning pregnancy by subtracting nine months. Age- (in five year intervals), parity- and urban/rural-stratified pregnancy rates were then calculated based upon the number of pregnancies occurring and person-years of exposure in each of these strata. The EIR in the model of P. falciparum transmission, using the was then calibrated so that prevalence by slide microscopy in 2-10 year-olds in the model matched that estimated by the Malaria Atlas Project (MAP) for each 5km^2^ pixel across Africa.

The reproductive histories of 100,000 women were then simulated for each pixel beginning at the age of 15, with the rates at which they become pregnant determined by the estimated age- and parity-specific pregnancy rates within the country and whether they were from a rural or urban setting (a probability for each pixel assigned as a function of population density as previously described). When a woman becomes pregnant within the model the rate at which she becomes pregnant again is then determined by the age-specific rate in women of the next parity, allowing for a minimum gap of a year between pregnancies. If a woman reaches the end of one 5-year age interval, the rate of next pregnancy is then determined by the parity-specific rate in the next 5-year age interval. The woman’s rate of pregnancy drops to zero when she passes the age of 50, the last year fertility data is collected in population-based surveys.

Each woman is also assigned one of the five transmission intensity strata within each setting according to the proportion of the population in each strata (as determined according to the deterministic approximation to equation S7). Whether she has peripheral parasitaemia (patent or sub-patent) at the beginning of each pregnancy is then determined by the proportion of women in her age and transmission strata who are similarly infected. The timing and frequency of peripheral infection during pregnancy is also simulated according to the rate of infection in her age and transmission strata.

**Placental infection, pregnancy-specific immunity and malaria-associated risk of LBW**

We assume pregnant women acquire and clear infection at the same rate as their non-pregnant counterparts until the 12^th^ week of gestation, around the time maternal blood first begins flowing into the intervillous space of the placenta. We then assume that, provided infection is sufficiently dense to sustain infection for a further week, in the absence of any immunity due to exposure to placental infection in prior pregnancies, the parasite will have been able to sequester within the placenta and begin replicating, often to very high densities. Once the parasite has sequestered it progresses through three histologically relevant stages of infection (see Figure S2-2): acute infection (defined as visible parasitisation within the intervillous space but little or none of the haemozoin pigment trapped within fibrin deposits or monocyte-marcophages infilitrates indicative of prolonged parasite digestion and an inflammatory innate immune reponse), chronic infection (defined as both the present of parasites and pigment) and past infection (no parasites are visible but pigment remains).


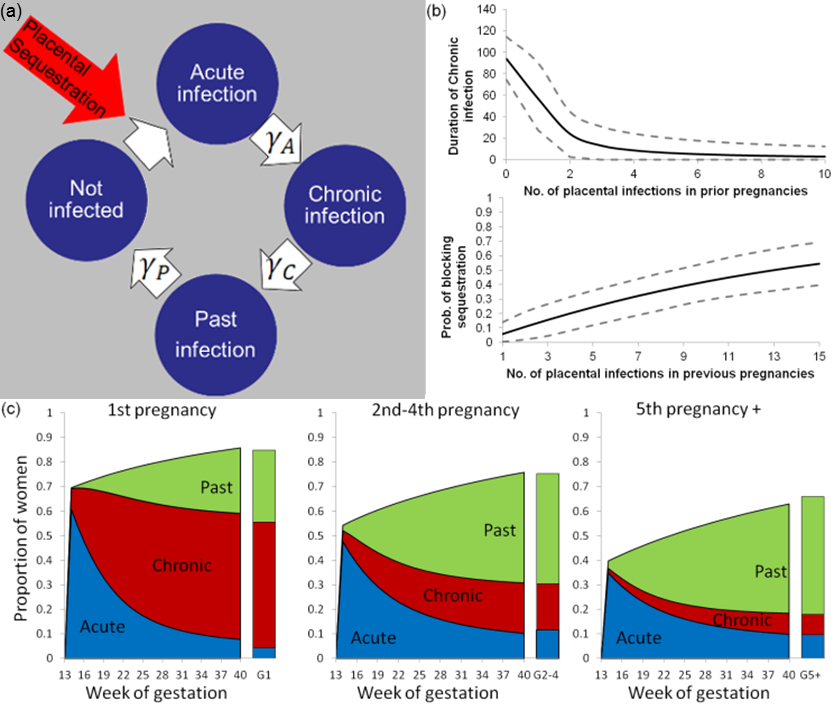


**Figure S2-2|The model of placental infection.** (a) Schematic of the progression of placental infection with peripheral infection when maternal blood is flowing into the placenta. (b) Estimates of the functional forms of pregnancy-specific immunity to curtail the duration of chronic infection and the probability of detectable infection sequestering within the placenta (c) Estimates of the prevalence of each histologically relevant stage of infection throughout gestation by gravidity in Ifakara, Tanzania (taken from Walker et al [1]. Bars show the proportion at delivery within each gravidity class in the observed data.

The duration of these stages were then fitted to data on the prevalence of each stage by histology in primigravid women in the absence of pregnancy-specific interventions where there was contemporary information on the transmission intensity within the setting (e.g. Figure S2-2c). It was then demonstrated that the prevalence of these stages of infection at delivery in women of higher gravidity could only be replicated by incorporating some form of pregnancy-specific immunity acquired over successive pregnancies. The best fitting model of the function of immunity was one which prevented a proportion of infections from sequestering to any sufficient density to leave behind visible parasites. The fitted form of this sequestration-blocking immunity function was:

$$SB\left(x\right)=\frac{1}{\left(1+\left(\frac{x}{\xi}\right)^{v}\right)}$$

where $SB$ is the probability an infection is prevented from sequestering, $x$ is the number of times a woman has been exposed to placental infection in previous pregnancies and $\xi$ and $\nu$ are scale and shape parameters fitted to the placental histology data. Infections which did sequester in women were also estimated to be cleared more rapidly as a result of pregnancy-specific acquired adaptive immunity according to the following function:

$$\gamma_{C}(x)=\gamma_{C0}\left(1+\left(\frac{x}{\pi}\right)^{\psi}\right)$$

where $\gamma(x)$is the rate of progression through the chronic stage of infection, $\tau_{C}$the rate of progression in primigravidae and $\psi$ and $\pi$ are scale and shape parameters fitted to the placental histology data (see Table S2-2 for a full list of parameters for the model progression of placental infection).

**Model fitting**

Here we present a brief description of the model-fitting process (see [14] and [15] for full details of how the model was fitted and which models of parity-dependent immunity were considered). Following a literature review, the model was fitted to all available data linking the relationship between parity, the risk of placental infection by histological stage at delivery (acute, chronic and past) in the absence of interventions and the associated risk of LBW. This involved two large-scale studies – Kilifi, Kenya [16] and Ifakara Town, Tanzania [17,18]. The parameters of the underlying transmission model were held constant to their joint-posterior median values with the exception of the EIR within each setting which we gave an informative prior on the basis of contemporary entomology within each setting.

Within each setting, given an underlying prevalence of acute, chronic and past infection, $\mu_{A}^{i},\mu_{C}^{i}$ and $\mu_{P}^{i}$, within women of the $i$th parity, the probability of observing the number of acute, chronic and past infections ($n_{A}^{i}$, $n_{C}^{i}$ and $n_{P}^{i}$ respectively) from a sample size $N^{i}$ can be calculated using a multinomial distribution

.

Working within a Bayesian framework, the likelihood of any set of model parameters $\theta$ given the observed data (where $k$ indexes study site) is then

$$P\left(\theta|D\right)\propto\prod_{i,k} P\left(n_{A}^{i,k},n_{C}^{i,k},n_{P}^{i,k}|N^{i,k},\mu_{A}^{i,k},\mu_{C}^{i,k},\mu_{P}^{i,k}\right)P\left(\mu_{A}^{i,k},\mu_{C}^{i,k},\mu_{P}^{i,k}|\theta\right)P\left(\theta\right).$$

The joint posterior distribution of $\theta$ (the EIR within each setting and all parameters listed in Table S2-2 with the exception of $\beta$) was then estimated using Monte Carlo Markov Chain (MCMC) methods (see [14] for full details). The fit of the model to the data for various assunmptions about the nature and function of pregnancy-specific dependent immunity was then assessed and compared using the Deviance Information Criterion (DIC)[19], of which the model used in this analysis provided the lowest value.

**Supplementary Table S2-2 Fitted parameters for the model of placental infection**

| Symbol | Description | Posterior Median (with 95% credible interval) |
| --- | --- | --- |
| $1/\tau_{A}$ | Mean duration of acute infection (days) | 53.7 (41.2,65.2) |
| $1/\tau_{C}$ | Mean duration of chronic infection (days) | 94.2 (73.5,118.1) |
| $\tau_{P}$ | Rate of progression through past stage placental infection (1/day) | 0.0002 (0.0001,0.0012) |
| $\nu$ | Power parameter of infection blocking immunity | 1.01 (0.51,2.36) |
| $\xi$ | Offset power of infection blocking immunity | 14.5. (8.3,37.4) |
| $\psi$ | Power parameter of faster clearance of chronic infection | 2.23 (0.59,9.68) |
| $\pi$ | Offset parameter of faster clearance of chronic infection | 1.16 (0.31,1.98) |
| $\beta$ | Additional hazard of LBW per day with chronic infection | 0.002 (0.001, 0.003) |

We then defined a risk of LBW due to malaria, $r_{ij}$, in a particular setting $i$ and parity $j$, dependent upon the duration of the chronic stage of infection, 1/$\gamma_{c}$ (which is likely to be an indication of the magnitude and duration of both the infection within the placenta and the ensuing inflammatory immune response) using the following equation:

$$r_{ij}\left( \gamma_{c},\beta\right)=1-(1-\rho_{ij})e^{-\left( \beta/\gamma_{c} \right)}$$

where $\rho_{ij}$ is the underlying risk of non-malaria-attributable LBW in women in setting $i$ with parity $j$ and $\beta$ is the hazard of LBW associated with a day with chronic infection.

Using the joint posterior distribution of the model parameters $\theta$ obtained from fitting the model to $D$, the observed patterns of placental infection by parity in each transmission setting it was possible to calculate $P(H_{ij}|D)=\int_{\theta} {P(H}_{ij}|x)P(\theta=x|D)dx$ the estimated joint posterior distribution of $H_{ij}$, the duration of chronic-stage infection of women of parity $j$ and infection category $i$. $P\left( L_{ij} | N_{ij},H_{ij},D \right)$, the likelihood of the number of LBW deliveries in each infection and parity specific category, $L_{ij}$, from the number of observations in that category, $N_{ij},$ can then be calculated as

$$P\left( L_{ij} | N_{ij},H_{ij},D,\beta\right)=\int_{H_{ij}} P\left( L_{ij} | N_{ij},\gamma_{c},\beta\right)P\left( H_{ij}=\gamma_{c}|D \right)dh=\int_{H_{ij}} \left( r_{ij}(\gamma_{c},\beta) \right)^{L_{ij}}\left( 1-r_{ij}(\gamma_{c,\beta}) \right)^{N_{ij}-L_{ij}}P\left( \gamma_{c}|D \right)d\gamma_{c}$$

Which was then fitted to the data using MCMC (see [15] for full details).

The assumption that malaria-attributable risk depends on the duration of chronic stage infection (defined as the presence of infection with histologically visible inflammatory immune response) was one of many tested (see [15] for full details). We found that this model above (where the risk of LBW depends upon the duration of infection following the initiation of a visible inflammatory immune response (i.e. the duration of ‘chronic’ infection) provided the best fit to the data both visually and measured by DIC. This model was able to replicate patterns of LBW by stage of infection and parity in the absence of intervention in Kilifi, Kenya and Ifakara, Tanzania substantially better than assuming a homogenous risk of LBW associated with placental infection or incorporating risks associated with any other stage (acute or past). It also supports the suggestion that LBW due to malaria in pregnancy is caused by a prolonged inflammatory immune response in women who are yet to develop immunity to placental parasites [20].

The uncertainty in our estimates of the risk and burden of malaria in pregnancy in the absence of intervention were then captured by the 95% credible intervals obtained by simulating the model for 10000 draws of the joint posterior distribution for the parameters listed in Table S1-2. These were then applied to the maps of malaria prevalence representing the highest and lowest estimates of overall prevalence from 100 realisations of the joint posterior of the map of prevalence across Africa in 2000 and 2015 as estimated by the Malaria Atlas Project [21] in an attempt to be conservative with respect to the joint posterior distribution of these two sources of uncertainty. For estimating the percentage change in risk and burden we used the range of these values as estimated from the joint posterior distribution of the spatial and temporal uncertainty inherent in these estimates.

**References**

1. Walker PGT, Griffin JT, Cairns M, Rogerson SJ, van Eijk AM, ter Kuile F, et al. A model of parity-dependent immunity to placental malaria. Nat Commun. 2013;4: 1609. doi:10.1038/ncomms2605

2. Walker PGT, ter Kuile FO, Garske T, Menendez C, Ghani AC. Estimated risk of placental infection and low birthweight attributable to Plasmodium falciparum malaria in Africa in 2010: a modelling study. Lancet Glob Heal. 2014;2: e460–7. doi:10.1016/S2214-109X(14)70256-6

3. Griffin JT, Hollingsworth TD, Okell LC, Churcher TS, White M, Hinsley W, et al. Reducing Plasmodium falciparum malaria transmission in Africa: a model-based evaluation of intervention strategies. PLoS Med. 2010;7. doi:10.1371/journal.pmed.1000324

4. Walker PGT, ter Kuile FO, Garske T, Menendez C, Ghani AC. Estimates of the risk of placental infection and burden of low birthweight attributable to P. falciparum malaria in Africa in 2010. PLoS Med. 2013;

5. EYLES DE, YOUNG MD. The duration of untreated or inadequately treated Plasmodium falciparum infections in the human host. Journal Natl Malar Soc. 1951;10: 327–36. Available: http://www.ncbi.nlm.nih.gov/pubmed/14908561

6. Molineaux L, Gramacia G. The Garki Project: research on the epidemiology and control of malaria in the Sudan savanna of West Africa. Geneva: World Health Organization; 1980.

7. WHO. World Malaria Report 2014. World Health Organization; 2014; Available: http://www.who.int/malaria/publications/world_malaria_report_2014/en/

8. Zwang J, Ashley EA, Karema C, D’Alessandro U, Smithuis F, Dorsey G, et al. Safety and Efficacy of Dihydroartemisinin-Piperaquine in Falciparum Malaria: A Prospective Multi-Centre Individual Patient Data Analysis. Diemert DJ, editor. PLoS One. 2009;4: e6358. doi:10.1371/journal.pone.0006358

9. MILLER MJ. Observations on the natural history of malaria in the semi-resistant West African. Trans R Soc Trop Med Hyg. 1958;52: 152–68. Available: http://www.ncbi.nlm.nih.gov/pubmed/13543904

10. FALK N, MAIRE N, SAMA W, OWUSU-AGYEI S, SMITH T, BECK H-P, et al. COMPARISON OF PCR-RFLP AND GENESCAN-BASED GENOTYPING FOR ANALYZING INFECTION DYNAMICS OF PLASMODIUM FALCIPARUM. Am J Trop Med Hyg. 2006;74: 944–950. Available: http://www.ajtmh.org/content/74/6/944.long

11. Gu W, Mbogo CM, Githure JI, Regens JL, Killeen GF, Swalm CM, et al. Low recovery rates stabilize malaria endemicity in areas of low transmission in coastal Kenya. Acta Trop. 2003;86: 71–81. Available: http://www.ncbi.nlm.nih.gov/pubmed/12711106

12. Killeen GF, McKenzie FE, Foy BD, Schieffelin C, Billingsley PF, Beier JC. A simplified model for predicting malaria entomologic inoculation rates based on entomologic and parasitologic parameters relevant to control. Am J Trop Med Hyg. 2000;62: 535–44. Available: http://www.pubmedcentral.nih.gov/articlerender.fcgi?artid=2483339&tool=pmcentrez&rendertype=abstract

13. Gillies MT, Wilkes TJ. A study of the age-composition of populations of Anopheles gambiae Giles and A. funestus Giles in North-Eastern Tanzania. Bull Entomol Res. 1965;56: 237–62. Available: http://www.ncbi.nlm.nih.gov/pubmed/5854754

14. Walker PGT, Griffin JT, Cairns M, Rogerson SJ, van Eijk AM, Ter Kuile F, et al. A model of parity-dependent immunity to placental malaria. Nat Commun. 2013;4: 1609. doi:10.1038/ncomms2605

15. Walker PGT, ter Kuile FO, Garske T, Menendez C, Ghani AC. Estimated risk of placental infection and low birthweight attributable to Plasmodium falciparum malaria in Africa in 2010: a modelling study. Lancet Glob Heal. 2014;2: e460–7. doi:10.1016/S2214-109X(14)70256-6

16. Shulman C, Marshall T, Dorman E, Bulmer J, Cutts F, Peshu N, et al. Malaria in pregnancy: adverse effects on haemoglobin levels and birthweight in primigravidae and multigravidae. Trop Med Int Heal. Wiley Online Library; 2001;6: 770–778. Available: http://www.ncbi.nlm.nih.gov/pubmed/11679125

17. Ismail MR, Ordi J, Menendez C, Ventura PJ, Aponte JJ, Kahigwa E, et al. Placental pathology in malaria: a histological, immunohistochemical, and quantitative study. Hum Pathol. 2000;31: 85–93. Available: http://www.ncbi.nlm.nih.gov/pubmed/10665918

18. Menendez C, Ordi J, Ismail MR, Ventura PJ, Aponte JJ, Kahigwa E, et al. The impact of placental malaria on gestational age and birth weight. J Infect Dis. 2000;181: 1740–5. doi:10.1086/315449

19. Spiegelhalter DJ, Best NG, Carlin BP, van der Linde A. Bayesian measures of model complexity and fit. J R Stat Soc Ser B (Statistical Methodol. 2002;64: 583–639. doi:10.1111/1467-9868.00353

20. Rogerson SJ, Mwapasa V, Meshnick SR. Malaria in pregnancy: linking immunity and pathogenesis to prevention. Am J Trop Med Hyg. 2007;77: 14–22. Available: http://www.ncbi.nlm.nih.gov/pubmed/18165470

21. Bhatt S, Weiss DJ, Cameron E, Bisanzio D, Mappin B, Dalrymple U, et al. The effect of malaria control on Plasmodium falciparum in Africa between 2000 and 2015. Nature. Nature Publishing Group; 2015;526: 207–211. doi:10.1038/nature15535
