# Supplementary material for: Estimated impact on birth weight of scaling up intermittent preventive treatment of malaria in pregnancy given sulphadoxine-pyrimethamine resistance in Africa: A mathematical model
Source: PLoS Med. 2017 Feb 28;14(2):e1002243. doi: 10.1371/journal.pmed.1002243 (PMC5330448; doi:10.1371/journal.pmed.1002243)
Supplement: S3 Appendix — (DOCX) [file pmed.1002243.s003.docx]

**Estimated Impact on Birth Weight of Intermittent Preventive Treatment for Malaria in Pregnancy Given Sulphadoxine-Pyrimethamine Resistance in Africa: a Mathematical Model**

**Text S3: Estimates of Impact by country, resistance strata and gravidity.**

**Table S3-1. Total Number of Women of Child-bearing age (1000s) by SP resistance stratum**

|  |  | **Total number of women of child-bearing age, by SP resistance stratum** | | | | | | | | | |
| --- | --- | --- | --- | --- | --- | --- | --- | --- | --- | --- | --- |
|  | Total WOCBA Population | IPTp-SP fully effective |  | IPTp-SP effective |  | SP at risk (1) |  | SP at risk(2) |  | Super-resistant |  |
|  |  | N | % | N | % | N | % | N | % | N | % |
| Burkina Faso | 4,958.8 | 4,958.8 | 100 | 0 | 0 | 0 | 0 | 0 | 0 | 0 | 0 |
| Benin | 2,863.0 | 2,863.0 | 100 | 0 | 0 | 0 | 0 | 0 | 0 | 0 | 0 |
| Cote d'Ivoire | 6,041.3 | 6,041.3 | 100 | 0 | 0 | 0 | 0 | 0 | 0 | 0 | 0 |
| The Gambia | 601.1 | 601.1 | 100 | 0 | 0 | 0 | 0 | 0 | 0 | 0 | 0 |
| Ghana | 8,171.4 | 8,171.4 | 100 | 0 | 0 | 0 | 0 | 0 | 0 | 0 | 0 |
| Guinea | 3,356.7 | 3,356.7 | 100 | 0 | 0 | 0 | 0 | 0 | 0 | 0 | 0 |
| Guinea-Bissau | 522.5 | 522.5 | 100 | 0 | 0 | 0 | 0 | 0 | 0 | 0 | 0 |
| Liberia | 1,145.9 | 1,145.9 | 100 | 0 | 0 | 0 | 0 | 0 | 0 | 0 | 0 |
| Mali | 3,826.4 | 3,826.4 | 100 | 0 | 0 | 0 | 0 | 0 | 0 | 0 | 0 |
| Mauritania | 1,083.0 | 1,083.0 | 100 | 0 | 0 | 0 | 0 | 0 | 0 | 0 | 0 |
| Niger | 4,148.1 | 4,148.1 | 100 | 0 | 0 | 0 | 0 | 0 | 0 | 0 | 0 |
| Nigeria | 45,543.5 | 45,543.5 | 100 | 0 | 0 | 0 | 0 | 0 | 0 | 0 | 0 |
| Sierra Leone | 2,142.3 | 2,142.3 | 100 | 0 | 0 | 0 | 0 | 0 | 0 | 0 | 0 |
| Senegal | 4,342.4 | 4,342.4 | 100 | 0 | 0 | 0 | 0 | 0 | 0 | 0 | 0 |
| Togo | 2,092.3 | 2,092.3 | 100 | 0 | 0 | 0 | 0 | 0 | 0 | 0 | 0 |
| **Total WARN** | 90,838.4 | 90,838.4 | 100 | 0 | 0 | 0 | 0 | 0 | 0 | 0 | 0 |
|  |  |  |  |  |  |  |  |  |  |  |  |
| Angola | 4,038.3 | 3,770.9 | 93.4 | 267.4 | 6.6 | 0 | 0 | 0 | 0 | 0 | 0 |
| C. African Rep. | 1,585.7 | 1,322.7 | 83.4 | 263.0 | 16.6 | 0 | 0 | 0 | 0 | 0 | 0 |
| Cameroon | 6,355.6 | 6,355.6 | 100 | 0 | 0 | 0 | 0 | 0 | 0 | 0 | 0 |
| Chad | 3,388.7 | 3,196.6 | 94.3 | 192.0 | 5.7 | 0 | 0 | 0 | 0 | 0 | 0 |
| D.R. Congo | 20,816.4 | 8,176.7 | 39.3 | 9,458.3 | 45.4 | 63.0 | 0.3 | 1,107.2 | 5.3 | 2,011.2 | 9.7 |
| Eq. Guinea | 203.7 | 203.7 | 100 | 0 | 0 | 0 | 0 | 0 | 0 | 0 | 0 |
| Gabon | 542.2 | 542.2 | 100 | 0 | 0 | 0 | 0 | 0 | 0 | 0 | 0 |
| Rep. of Congo | 1,341.2 | 1,341.2 | 100 | 0 | 0 | 0 | 0 | 0 | 0 | 0 | 0 |
| **Total CARN** | 38,271.8 | 24,909.6 | 65.1 | 10,180.8 | 26.6 | 63.0 | 0.2 | 1,107.2 | 2.9 | 2,011.2 | 5.3 |
|  |  |  |  |  |  |  |  |  |  |  |  |
| Burundi | 2,954.7 | 0 | 0 | 375.2 | 12.7 | 0 | 0 | 13.8 | 0.5 | 2,565.8 | 86.8 |
| Djibouti | 136.3 | 0 | 0 | 136.3 | 100 | 0 | 0 | 0 | 0 | 0 | 0 |
| Eritrea | 1,507.1 | 0 | 0 | 1,482.3 | 98.4 | 24.8 | 1.6 | 0 | 0 | 0 | 0 |
| Ethiopia | 22,264.5 | 0 | 0 | 6,451.1 | 29.0 | 15,813.4 | 71.0 | 0 | 0 | 0 | 0 |
| Kenya | 10,166.0 | 0 | 0 | 156.1 | 1.5 | 8,925.2 | 87.8 | 914.5 | 9.0 | 170.3 | 1.7 |
| Rwanda | 2,622.6 | 0 | 0 | 0 | 0 | 0 | 0 | 0 | 0 | 2,622.6 | 100 |
| Somalia | 3,003.9 | 0 | 0 | 3,002.9 | 100 | 1.0 | 0 | 0 | 0 | 0 | 0 |
| S. Sudan | 2,382.5 | 0 | 0 | 1,920.1 | 80.6 | 462.3 | 19.4 | 0 | 0 | 0 | 0 |
| Sudan | 11,343.3 | 0 | 0 | 11,311.7 | 99.7 | 31.6 | 0.3 | 0 | 0 | 0 | 0 |
| Uganda | 9,536.4 | 0 | 0 | 0 | 0 | 4,583.9 | 48.1 | 2,951.2 | 30.9 | 2,001.3 | 21.0 |
| **Total EARN** | 65,917.5 | 0 | 0 | 24,835.8 | 37.7 | 29,842.2 | 45.3 | 3,879.5 | 5.9 | 7,359.9 | 11.2 |
|  |  |  |  |  |  |  |  |  |  |  |  |
| Botswana | 307.9 | 0 | 0 | 307.9 | 100 | 0 | 0 | 0 | 0 | 0 | 0 |
| Madagascar | 4,971.9 | 4,971.9 | 100 | 0 | 0 | 0 | 0 | 0 | 0 | 0 | 0 |
| Malawi | 4,570.1 | 0 | 0 | 0 | 0 | 4,570.1 | 100 | 0 | 0 | 0 | 0 |
| Mozambique | 7,385.9 | 0 | 0 | 6,047.1 | 81.9 | 1,338.8 | 18.1 | 0 | 0 | 0 | 0 |
| Namibia | 522.1 | 376.7 | 72.2 | 145.3 | 27.8 | 0 | 0 | 0 | 0 | 0 | 0 |
| South Africa | 2,186.7 | 0 | 0 | 2,186.7 | 100 | 0 | 0 | 0 | 0 | 0 | 0 |
| Swaziland | 12.9 | 0 | 0 | 12.9 | 100 | 0 | 0 | 0 | 0 | 0 | 0 |
| Tanzania | 12,104.0 | 0 | 0 | 5,026.4 | 41.5 | 3,916.8 | 32.4 | 1,975.7 | 16.3 | 1,185.1 | 9.8 |
| Zambia | 3,749.3 | 0 | 0 | 3,009.6 | 80.3 | 739.6 | 19.7 | 0 | 0 | 0 | 0 |
| Zimbabwe | 4,171.5 | 0 | 0 | 4,171.1 | 100 | 0.4 | 0 | 0 | 0 | 0 | 0 |
| **Total SARN** | 39,982.2 | 5,348.6 | 13.4 | 20,907.0 | 52.3 | 10,565.8 | 26.4 | 1,975.7 | 4.9 | 1,185.1 | 3.0 |
|  |  |  |  |  |  |  |  |  |  |  |  |
| **Total SSA** | 235,009.9 | 121,096.6 | 51.5 | 55,923.6 | 23.8 | 40,471.0 | 17.2 | 6,962.4 | 3.0 | 10,556.3 | 4.5 |

**Table S3-2. Number of pregnant women (1000s), by Gravidity**

|  |  | **Number of pregnant women, by gravidity** | | | | | | | | | |
| --- | --- | --- | --- | --- | --- | --- | --- | --- | --- | --- | --- |
|  | Total No. Pregnant Women | Primi-gravid |  | Secundi-gravid |  | Gravidity  3 |  | Gravidity  4 |  | Gravidity 5+ |  |
|  |  | N | % | N | % | N | % | N | % | N | % |
| Burkina Faso | 708.0 | 132.2 | 18.7 | 123.9 | 17.5 | 112.8 | 15.9 | 101.6 | 14.3 | 237.5 | 33.5 |
| Benin | 373.4 | 79.1 | 21.2 | 74.7 | 20 | 67.7 | 18.1 | 56.7 | 15.2 | 95.3 | 25.5 |
| Cote d'Ivoire | 701.6 | 157.4 | 22.4 | 135.1 | 19.3 | 115.5 | 16.5 | 93.5 | 13.3 | 200.1 | 28.5 |
| The Gambia | 79.7 | 16.1 | 20.2 | 14.0 | 17.5 | 12.1 | 15.2 | 10.5 | 13.2 | 27.0 | 33.9 |
| Ghana | 775.8 | 204.7 | 26.4 | 178.0 | 22.9 | 142.4 | 18.4 | 98.8 | 12.7 | 152.0 | 19.6 |
| Guinea | 429.4 | 89.1 | 20.8 | 81.5 | 19.0 | 70.2 | 16.3 | 58.8 | 13.7 | 129.7 | 30.2 |
| Guinea-Bissau | 67.3 | 13.9 | 20.6 | 12.4 | 18.4 | 10.9 | 16.2 | 8.7 | 12.9 | 21.5 | 31.9 |
| Liberia | 155.3 | 31.6 | 20.3 | 30 | 19.3 | 25.9 | 16.7 | 21.0 | 13.5 | 46.8 | 30.1 |
| Mali | 600.2 | 104.8 | 17.5 | 100.5 | 16.7 | 94.5 | 15.7 | 85.9 | 14.3 | 214.6 | 35.7 |
| Mauritania | 167.8 | 29.6 | 17.6 | 28.4 | 16.9 | 26.6 | 15.9 | 24.1 | 14.4 | 59.1 | 35.2 |
| Niger | 763.9 | 113.9 | 14.9 | 109.5 | 14.3 | 102.7 | 13.4 | 95.0 | 12.4 | 342.9 | 44.9 |
| Nigeria | 6,091.4 | 1,200 | 19.7 | 1,133.6 | 18.6 | 1,009.9 | 16.6 | 861.0 | 14.1 | 1,887.0 | 31.0 |
| Sierra Leone | 255.3 | 57.5 | 22.5 | 51.7 | 20.3 | 44.0 | 17.2 | 35.3 | 13.8 | 66.8 | 26.2 |
| Senegal | 535.8 | 114.4 | 21.3 | 100.1 | 18.7 | 88.2 | 16.5 | 66.9 | 12.5 | 166.2 | 31.0 |
| Togo | 290.1 | 57.9 | 20.0 | 54.6 | 18.8 | 49.6 | 17.1 | 41.1 | 14.2 | 86.8 | 29.9 |
| **Total WARN** | 11,995.1 | 2,401.9 | 20.0 | 2,227.9 | 18.6 | 1,973.1 | 16.4 | 1,659.0 | 13.8 | 3,733.2 | 31.1 |
|  |  |  |  |  |  |  |  |  |  |  |  |
| Angola | 673.8 | 110.0 | 16.3 | 104.3 | 15.5 | 98.7 | 14.6 | 88.5 | 13.1 | 272.3 | 40.4 |
| C. African Rep. | 250.2 | 42.7 | 17.1 | 39.9 | 16.0 | 36.9 | 14.7 | 33.3 | 13.3 | 97.4 | 38.9 |
| Cameroon | 822.4 | 167.7 | 20.4 | 151.7 | 18.4 | 133.2 | 16.2 | 107.8 | 13.1 | 262.0 | 31.9 |
| Chad | 530.1 | 90.9 | 17.1 | 84.7 | 16.0 | 79.6 | 15.0 | 73.6 | 13.9 | 201.4 | 38.0 |
| D.R. Congo | 3,469.0 | 561.6 | 16.2 | 527.9 | 15.2 | 489.6 | 14.1 | 450 | 13.0 | 1,439.9 | 41.5 |
| Eq. Guinea | 25.3 | 5.5 | 21.8 | 4.7 | 18.7 | 3.9 | 15.4 | 3.3 | 13.1 | 7.8 | 31.0 |
| Gabon | 84.7 | 14.8 | 17.5 | 13.5 | 15.9 | 11.9 | 14.1 | 10.9 | 12.8 | 33.6 | 39.7 |
| Rep. of Congo | 178.4 | 36.8 | 20.7 | 34.5 | 19.4 | 30.3 | 17.0 | 25.2 | 14.1 | 51.6 | 28.9 |
| **Total CARN** | 6,033.9 | 1,030.0 | 17.1 | 961.1 | 15.9 | 884.0 | 14.7 | 792.6 | 13.1 | 2,366.0 | 39.2 |
|  |  |  |  |  |  |  |  |  |  |  |  |
| Burundi | 397.2 | 80.3 | 20.2 | 72.2 | 18.2 | 69.3 | 17.5 | 57.1 | 14.4 | 118.2 | 29.8 |
| Djibouti | 8.5 | 3.2 | 37.7 | 2.1 | 25.0 | 1.4 | 16.2 | 0.8 | 9.4 | 1.0 | 11.7 |
| Eritrea | 141.7 | 37.5 | 26.4 | 29.4 | 20.7 | 23.2 | 16.4 | 17.7 | 12.5 | 34.0 | 24.0 |
| Ethiopia | 2,229.6 | 559.5 | 25.1 | 451.0 | 20.2 | 365.9 | 16.4 | 286.0 | 12.8 | 567.3 | 25.4 |
| Kenya | 1,008.8 | 268.5 | 26.6 | 242.9 | 24.1 | 186.0 | 18.4 | 125.1 | 12.4 | 186.2 | 18.5 |
| Rwanda | 249.1 | 71.5 | 28.7 | 68.7 | 27.6 | 46.0 | 18.5 | 31.2 | 12.5 | 31.7 | 12.7 |
| Somalia | 314.0 | 76.5 | 24.4 | 63.7 | 20.3 | 52.3 | 16.7 | 40.8 | 13.0 | 80.6 | 25.7 |
| S. Sudan | 345.8 | 58.7 | 17.0 | 53.5 | 15.5 | 50.8 | 14.7 | 44.3 | 12.8 | 138.5 | 40.1 |
| Sudan | 1,305.4 | 277.5 | 21.3 | 230.4 | 17.6 | 198.7 | 15.2 | 161.7 | 12.4 | 437.1 | 33.5 |
| Uganda | 1,265.3 | 255.2 | 20.2 | 239.0 | 18.9 | 213.9 | 16.9 | 181.6 | 14.4 | 375.6 | 29.7 |
| **Total EARN** | 7,265.3 | 1,688.5 | 23.2 | 1,452.9 | 20.0 | 1,207.5 | 16.6 | 946.3 | 13.0 | 1,970.2 | 27.1 |
|  |  |  |  |  |  |  |  |  |  |  |  |
| Botswana | 32.7 | 8.4 | 25.7 | 7.7 | 23.6 | 6.4 | 19.5 | 4.3 | 13.2 | 5.9 | 18.1 |
| Madagascar | 523.8 | 134.9 | 25.8 | 123.6 | 23.6 | 100 | 19.1 | 71.8 | 13.7 | 93.4 | 17.8 |
| Malawi | 569.4 | 122.7 | 21.5 | 117.9 | 20.7 | 102.9 | 18.1 | 82.1 | 14.4 | 143.9 | 25.3 |
| Mozambique | 1,312.9 | 204.8 | 15.6 | 197.9 | 15.1 | 183.5 | 14.0 | 170.8 | 13.0 | 555.9 | 42.3 |
| Namibia | 56.5 | 14.1 | 25.0 | 13.0 | 23.0 | 10.5 | 18.6 | 7.3 | 12.8 | 11.7 | 20.6 |
| South Africa | 251.0 | 59.2 | 23.6 | 53.5 | 21.3 | 43.8 | 17.5 | 32.2 | 12.8 | 62.4 | 24.8 |
| Swaziland | 1.3 | 0.3 | 26.3 | 0.3 | 23.8 | 0.2 | 18.6 | 0.2 | 13.3 | 0.2 | 18.0 |
| Tanzania | 1,588.3 | 323.9 | 20.4 | 295.8 | 18.6 | 255.1 | 16.1 | 211.9 | 13.3 | 501.6 | 31.6 |
| Zambia | 529.3 | 101.8 | 19.2 | 94.4 | 17.8 | 83.3 | 15.7 | 70.8 | 13.4 | 179.0 | 33.8 |
| Zimbabwe | 453.1 | 114.2 | 25.2 | 105.5 | 23.3 | 88.7 | 19.6 | 61.4 | 13.5 | 83.3 | 18.4 |
| **Total SARN** | 5,318.3 | 1,084.3 | 20.4 | 1,009.6 | 19.0 | 874.5 | 16.4 | 712.6 | 13.4 | 1,637.3 | 30.8 |
|  |  |  |  |  |  |  |  |  |  |  |  |
| **Total SSA** | 30,612.5 | 6,204.8 | 20.3 | 5,651.5 | 18.5 | 4,939.1 | 16.1 | 4,110.5 | 13.4 | 9,706.7 | 31.7 |

**Table S3-3. Number of pregnant women (1000s) by SP resistance stratum**

|  |  | **Number of pregnant women, by SP resistance stratum** | | | | | | | | | |
| --- | --- | --- | --- | --- | --- | --- | --- | --- | --- | --- | --- |
|  | Total No. Pregnant Women | IPTp-SP fully effective |  | IPTp-SP effective |  | SP at risk (1) |  | SP at risk(2) |  | Super-resistant |  |
|  |  | N | % | N | % | N | % | N | % | N | % |
| Burkina Faso | 708.0 | 708.0 | 100 | 0 | 0 | 0 | 0 | 0 | 0 | 0 | 0 |
| Benin | 373.4 | 373.4 | 100 | 0 | 0 | 0 | 0 | 0 | 0 | 0 | 0 |
| Cote d'Ivoire | 701.6 | 701.6 | 100 | 0 | 0 | 0 | 0 | 0 | 0 | 0 | 0 |
| The Gambia | 79.7 | 79.7 | 100 | 0 | 0 | 0 | 0 | 0 | 0 | 0 | 0 |
| Ghana | 775.8 | 775.8 | 100 | 0 | 0 | 0 | 0 | 0 | 0 | 0 | 0 |
| Guinea | 429.4 | 429.4 | 100 | 0 | 0 | 0 | 0 | 0 | 0 | 0 | 0 |
| Guinea-Bissau | 67.3 | 67.3 | 100 | 0 | 0 | 0 | 0 | 0 | 0 | 0 | 0 |
| Liberia | 155.3 | 155.3 | 100 | 0 | 0 | 0 | 0 | 0 | 0 | 0 | 0 |
| Mali | 600.2 | 600.2 | 100 | 0 | 0 | 0 | 0 | 0 | 0 | 0 | 0 |
| Mauritania | 167.8 | 167.8 | 100 | 0 | 0 | 0 | 0 | 0 | 0 | 0 | 0 |
| Niger | 763.9 | 763.9 | 100 | 0 | 0 | 0 | 0 | 0 | 0 | 0 | 0 |
| Nigeria | 6,091.4 | 6,091.4 | 100 | 0 | 0 | 0 | 0 | 0 | 0 | 0 | 0 |
| Sierra Leone | 255.3 | 255.3 | 100 | 0 | 0 | 0 | 0 | 0 | 0 | 0 | 0 |
| Senegal | 535.8 | 535.8 | 100 | 0 | 0 | 0 | 0 | 0 | 0 | 0 | 0 |
| Togo | 290.1 | 290.1 | 100 | 0 | 0 | 0 | 0 | 0 | 0 | 0 | 0 |
| **Total WARN** | 11,995.1 | 11,995.1 | 100 | 0 | 0 | 0 | 0 | 0 | 0 | 0 | 0 |
|  |  |  |  |  |  |  |  |  |  |  |  |
| Angola | 673.8 | 625.8 | 92.9 | 47.9 | 7.1 | 0 | 0 | 0 | 0 | 0 | 0 |
| C. African Rep. | 250.2 | 203.1 | 81.2 | 47.1 | 18.8 | 0 | 0 | 0 | 0 | 0 | 0 |
| Cameroon | 822.4 | 822.4 | 100 | 0 | 0 | 0 | 0 | 0 | 0 | 0 | 0 |
| Chad | 530.1 | 499.1 | 94.1 | 31.0 | 5.9 | 0 | 0 | 0 | 0 | 0 | 0 |
| D.R. Congo | 3,469.0 | 1,349.7 | 38.9 | 1,602.3 | 46.2 | 11.5 | 0.3 | 188.2 | 5.4 | 317.3 | 9.1 |
| Eq. Guinea | 25.3 | 25.3 | 100 | 0 | 0 | 0 | 0 | 0 | 0 | 0 | 0 |
| Gabon | 84.7 | 84.7 | 100 | 0 | 0 | 0 | 0 | 0 | 0 | 0 | 0 |
| Rep. of Congo | 178.4 | 178.4 | 100 | 0 | 0 | 0 | 0 | 0 | 0 | 0 | 0 |
| **Total CARN** | 6,033.9 | 3,788.5 | 62.8 | 1,728.4 | 28.6 | 11.5 | 0.2 | 188.2 | 3.1 | 317.3 | 5.3 |
|  |  |  |  |  |  |  |  |  |  |  |  |
| Burundi | 397.2 | 0 | 0 | 52.7 | 13.3 | 0 | 0 | 2.2 | 0.5 | 342.3 | 86.2 |
| Djibouti | 8.5 | 0 | 0 | 8.5 | 100 | 0 | 0 | 0 | 0 | 0 | 0 |
| Eritrea | 141.7 | 0 | 0 | 138.7 | 97.9 | 3.0 | 2.1 | 0 | 0 | 0 | 0 |
| Ethiopia | 2,229.6 | 0 | 0 | 642.8 | 28.8 | 1,586.8 | 71.2 | 0 | 0 | 0 | 0 |
| Kenya | 1,008.8 | 0 | 0 | 17.0 | 1.7 | 881.6 | 87.4 | 90.1 | 8.9 | 20 | 2.0 |
| Rwanda | 249.1 | 0 | 0 | 0 | 0 | 0 | 0 | 0 | 0 | 249.1 | 100 |
| Somalia | 314.0 | 0 | 0 | 313.9 | 100 | 0.1 | 0 | 0 | 0 | 0 | 0 |
| S. Sudan | 345.8 | 0 | 0 | 278.8 | 80.6 | 67.1 | 19.4 | 0 | 0 | 0 | 0 |
| Sudan | 1,305.4 | 0 | 0 | 1,300.5 | 99.6 | 4.8 | 0.4 | 0 | 0 | 0 | 0 |
| Uganda | 1,265.3 | 0 | 0 | 0 | 0 | 615.8 | 48.7 | 386.4 | 30.5 | 263.2 | 20.8 |
| **Total EARN** | 7,265.3 | 0 | 0 | 2,752.9 | 37.9 | 3,159.2 | 43.5 | 478.6 | 6.6 | 874.6 | 12.0 |
|  |  |  |  |  |  |  |  |  |  |  |  |
| Botswana | 32.7 | 0 | 0 | 32.7 | 100 | 0 | 0 | 0 | 0 | 0 | 0 |
| Madagascar | 523.8 | 523.8 | 100 | 0 | 0 | 0 | 0 | 0 | 0 | 0 | 0 |
| Malawi | 569.4 | 0 | 0 | 0 | 0 | 569.4 | 100 | 0 | 0 | 0 | 0 |
| Mozambique | 1,312.9 | 0 | 0 | 1,074.9 | 81.9 | 237.9 | 18.1 | 0 | 0 | 0 | 0 |
| Namibia | 56.5 | 39.9 | 70.6 | 16.6 | 29.4 | 0 | 0 | 0 | 0 | 0 | 0 |
| South Africa | 251.0 | 0 | 0 | 251.0 | 100 | 0 | 0 | 0 | 0 | 0 | 0 |
| Swaziland | 1.3 | 0 | 0 | 1.3 | 100 | 0 | 0 | 0 | 0 | 0 | 0 |
| Tanzania | 1,588.3 | 0 | 0 | 641.0 | 40.4 | 525.4 | 33.1 | 262.7 | 16.5 | 159.2 | 10 |
| Zambia | 529.3 | 0 | 0 | 412.2 | 77.9 | 117.1 | 22.1 | 0 | 0 | 0 | 0 |
| Zimbabwe | 453.1 | 0 | 0 | 453.1 | 100 | 0.1 | 0 | 0 | 0 | 0 | 0 |
| **Total SARN** | 5,318.3 | 563.7 | 10.6 | 2,882.9 | 54.2 | 1,449.9 | 27.3 | 262.7 | 4.9 | 159.2 | 3.0 |
|  |  |  |  |  |  |  |  |  |  |  |  |
| **Total SSA** | 30,612.5 | 16,347.3 | 53.4 | 7,364.2 | 24.1 | 4,620.6 | 15.1 | 929.5 | 3.0 | 1,351.1 | 4.4 |

**Table S3-4. Number of primigravid women (1000s) by SP resistance stratum**

|  |  | **Number of primigravidae, by SP resistance stratum** | | | | | | | | | |
| --- | --- | --- | --- | --- | --- | --- | --- | --- | --- | --- | --- |
|  | Total No. Primi-gravidae | IPTp-SP fully effective |  | IPTp-SP effective |  | SP at risk (1) |  | SP at risk(2) |  | Super-resistant |  |
|  |  | N | % | N | % | N | % | N | % | N | % |
| Burkina Faso | 132.2 | 132.2 | 100 | 0 | 0 | 0 | 0 | 0 | 0 | 0 | 0 |
| Benin | 79.1 | 79.1 | 100 | 0 | 0 | 0 | 0 | 0 | 0 | 0 | 0 |
| Cote d'Ivoire | 157.4 | 157.4 | 100 | 0 | 0 | 0 | 0 | 0 | 0 | 0 | 0 |
| The Gambia | 16.1 | 16.1 | 100 | 0 | 0 | 0 | 0 | 0 | 0 | 0 | 0 |
| Ghana | 204.7 | 204.7 | 100 | 0 | 0 | 0 | 0 | 0 | 0 | 0 | 0 |
| Guinea | 89.1 | 89.1 | 100 | 0 | 0 | 0 | 0 | 0 | 0 | 0 | 0 |
| Guinea-Bissau | 13.9 | 13.9 | 100 | 0 | 0 | 0 | 0 | 0 | 0 | 0 | 0 |
| Liberia | 31.6 | 31.6 | 100 | 0 | 0 | 0 | 0 | 0 | 0 | 0 | 0 |
| Mali | 104.8 | 104.8 | 100 | 0 | 0 | 0 | 0 | 0 | 0 | 0 | 0 |
| Mauritania | 29.6 | 29.6 | 100 | 0 | 0 | 0 | 0 | 0 | 0 | 0 | 0 |
| Niger | 113.9 | 113.9 | 100 | 0 | 0 | 0 | 0 | 0 | 0 | 0 | 0 |
| Nigeria | 1,200 | 1,200 | 100 | 0 | 0 | 0 | 0 | 0 | 0 | 0 | 0 |
| Sierra Leone | 57.5 | 57.5 | 100 | 0 | 0 | 0 | 0 | 0 | 0 | 0 | 0 |
| Senegal | 114.4 | 114.4 | 100 | 0 | 0 | 0 | 0 | 0 | 0 | 0 | 0 |
| Togo | 57.9 | 57.9 | 100 | 0 | 0 | 0 | 0 | 0 | 0 | 0 | 0 |
| Total WARN | 2,401.9 | 2,401.9 | 100 | 0 | 0 | 0 | 0 | 0 | 0 | 0 | 0 |
|  |  |  |  |  |  |  |  |  |  |  |  |
| Angola | 110 | 102.6 | 93.3 | 7.4 | 6.7 | 0 | 0 | 0 | 0 | 0 | 0 |
| C. African Rep. | 42.7 | 35.5 | 83.1 | 7.2 | 16.9 | 0 | 0 | 0 | 0 | 0 | 0 |
| Cameroon | 167.7 | 167.7 | 100 | 0 | 0 | 0 | 0 | 0 | 0 | 0 | 0 |
| Chad | 90.9 | 85.7 | 94.3 | 5.2 | 5.7 | 0 | 0 | 0 | 0 | 0 | 0 |
| D.R. Congo | 561.6 | 220.3 | 39.2 | 255.9 | 45.6 | 1.7 | 0.3 | 30 | 5.3 | 53.8 | 9.6 |
| Eq. Guinea | 5.5 | 5.5 | 100 | 0 | 0 | 0 | 0 | 0 | 0 | 0 | 0 |
| Gabon | 14.8 | 14.8 | 100 | 0 | 0 | 0 | 0 | 0 | 0 | 0 | 0 |
| Rep. of Congo | 36.8 | 36.8 | 100 | 0 | 0 | 0 | 0 | 0 | 0 | 0 | 0 |
| Total CARN | 1,030 | 668.9 | 64.9 | 275.7 | 26.8 | 1.7 | 0.2 | 30 | 2.9 | 53.8 | 5.2 |
|  |  |  |  |  |  |  |  |  |  |  |  |
| Burundi | 80.3 | 0 | 0 | 10.2 | 12.6 | 0 | 0 | 0.4 | 0.5 | 69.8 | 86.9 |
| Djibouti | 3.2 | 0 | 0 | 3.2 | 100 | 0 | 0 | 0 | 0 | 0 | 0 |
| Eritrea | 37.5 | 0 | 0 | 36.8 | 98.3 | 0.6 | 1.7 | 0 | 0 | 0 | 0 |
| Ethiopia | 559.5 | 0 | 0 | 162.0 | 28.9 | 397.5 | 71.1 | 0 | 0 | 0 | 0 |
| Kenya | 268.5 | 0 | 0 | 4.2 | 1.5 | 235.7 | 87.8 | 24.1 | 9.0 | 4.6 | 1.7 |
| Rwanda | 71.5 | 0 | 0 | 0 | 0 | 0 | 0 | 0 | 0 | 71.5 | 100 |
| Somalia | 76.5 | 0 | 0 | 76.5 | 100 | 0 | 0 | 0 | 0 | 0 | 0 |
| S. Sudan | 58.7 | 0 | 0 | 47.3 | 80.6 | 11.4 | 19.4 | 0 | 0 | 0 | 0 |
| Sudan | 277.5 | 0 | 0 | 276.7 | 99.7 | 0.8 | 0.3 | 0 | 0 | 0 | 0 |
| Uganda | 255.2 | 0 | 0 | 0 | 0 | 122.8 | 48.1 | 78.9 | 30.9 | 53.5 | 21.0 |
| Total EARN | 1,688.5 | 0 | 0 | 616.9 | 36.5 | 768.8 | 45.5 | 103.4 | 6.1 | 199.4 | 11.8 |
|  |  |  |  |  |  |  |  |  |  |  |  |
| Botswana | 8.4 | 0 | 0 | 8.4 | 100 | 0 | 0 | 0 | 0 | 0 | 0 |
| Madagascar | 134.9 | 134.9 | 100 | 0 | 0 | 0 | 0 | 0 | 0 | 0 | 0 |
| Malawi | 122.7 | 0 | 0 | 0 | 0 | 122.7 | 100 | 0 | 0 | 0 | 0 |
| Mozambique | 204.8 | 0 | 0 | 167.7 | 81.9 | 37.1 | 18.1 | 0 | 0 | 0 | 0 |
| Namibia | 14.1 | 10.2 | 71.9 | 4.0 | 28.1 | 0 | 0 | 0 | 0 | 0 | 0 |
| South Africa | 59.2 | 0 | 0 | 59.2 | 100 | 0 | 0 | 0 | 0 | 0 | 0 |
| Swaziland | 0.3 | 0 | 0 | 0.3 | 100 | 0 | 0 | 0 | 0 | 0 | 0 |
| Tanzania | 323.9 | 0 | 0 | 133.9 | 41.3 | 105.2 | 32.5 | 53.0 | 16.4 | 31.8 | 9.8 |
| Zambia | 101.8 | 0 | 0 | 81.6 | 80.2 | 20.2 | 19.8 | 0 | 0 | 0 | 0 |
| Zimbabwe | 114.2 | 0 | 0 | 114.2 | 100 | 0 | 0 | 0 | 0 | 0 | 0 |
| Total SARN | 1,084.3 | 145.1 | 13.4 | 569.2 | 52.5 | 285.2 | 26.3 | 53.0 | 4.9 | 31.8 | 2.9 |
|  |  |  |  |  |  |  |  |  |  |  |  |
| Total SSA | 6,204.8 | 3,215.9 | 51.8 | 1,461.8 | 23.6 | 1,055.8 | 17.0 | 186.4 | 3.0 | 285.0 | 4.6 |

**Table S3-5 Total number of infected pregnancies (1000s) by SP resistance stratum**

|  |  | **Number of infected pregnancies, by SP resistance stratum** | | | | | | | | | |
| --- | --- | --- | --- | --- | --- | --- | --- | --- | --- | --- | --- |
|  | Total No. infected pregnancies | IPTp-SP fully effective |  | IPTp-SP effective |  | SP at risk (1) |  | SP at risk(2) |  | Super-resistant |  |
|  |  | N | % | N | % | N | % | N | % | N | % |
| Burkina Faso | 409.2 | 409.2 | 100.0 | 0.0 | 0.0 | 0.0 | 0.0 | 0.0 | 0.0 | 0.0 | 0.0 |
| Benin | 159.0 | 159.0 | 100.0 | 0.0 | 0.0 | 0.0 | 0.0 | 0.0 | 0.0 | 0.0 | 0.0 |
| Cote d'Ivoire | 368.5 | 368.5 | 100.0 | 0.0 | 0.0 | 0.0 | 0.0 | 0.0 | 0.0 | 0.0 | 0.0 |
| The Gambia | 5.7 | 5.7 | 100.0 | 0.0 | 0.0 | 0.0 | 0.0 | 0.0 | 0.0 | 0.0 | 0.0 |
| Ghana | 350.3 | 350.3 | 100.0 | 0.0 | 0.0 | 0.0 | 0.0 | 0.0 | 0.0 | 0.0 | 0.0 |
| Guinea | 230.9 | 230.9 | 100.0 | 0.0 | 0.0 | 0.0 | 0.0 | 0.0 | 0.0 | 0.0 | 0.0 |
| Guinea-Bissau | 11.4 | 11.4 | 100.0 | 0.0 | 0.0 | 0.0 | 0.0 | 0.0 | 0.0 | 0.0 | 0.0 |
| Liberia | 64.0 | 64.0 | 100.0 | 0.0 | 0.0 | 0.0 | 0.0 | 0.0 | 0.0 | 0.0 | 0.0 |
| Mali | 339.7 | 339.7 | 100.0 | 0.0 | 0.0 | 0.0 | 0.0 | 0.0 | 0.0 | 0.0 | 0.0 |
| Mauritania | 19.1 | 19.1 | 100.0 | 0.0 | 0.0 | 0.0 | 0.0 | 0.0 | 0.0 | 0.0 | 0.0 |
| Niger | 289.3 | 289.3 | 100.0 | 0.0 | 0.0 | 0.0 | 0.0 | 0.0 | 0.0 | 0.0 | 0.0 |
| Nigeria | 2,707.1 | 2,707.1 | 100.0 | 0.0 | 0.0 | 0.0 | 0.0 | 0.0 | 0.0 | 0.0 | 0.0 |
| Sierra Leone | 105.9 | 105.9 | 100.0 | 0.0 | 0.0 | 0.0 | 0.0 | 0.0 | 0.0 | 0.0 | 0.0 |
| Senegal | 95.5 | 95.5 | 100.0 | 0.0 | 0.0 | 0.0 | 0.0 | 0.0 | 0.0 | 0.0 | 0.0 |
| Togo | 168.3 | 168.3 | 100.0 | 0.0 | 0.0 | 0.0 | 0.0 | 0.0 | 0.0 | 0.0 | 0.0 |
| **Total WARN** | 5,323.8 | 5,323.8 | 100.0 | 0.0 | 0.0 | 0.0 | 0.0 | 0.0 | 0.0 | 0.0 | 0.0 |
|  |  |  |  |  |  |  |  |  |  |  |  |
| Angola | 156.5 | 144.3 | 92.2 | 12.3 | 7.8 | 0.0 | 0.0 | 0.0 | 0.0 | 0.0 | 0.0 |
| C. African Rep. | 115.5 | 91.6 | 79.4 | 23.8 | 20.6 | 0.0 | 0.0 | 0.0 | 0.0 | 0.0 | 0.0 |
| Cameroon | 291.7 | 291.7 | 100.0 | 0.0 | 0.0 | 0.0 | 0.0 | 0.0 | 0.0 | 0.0 | 0.0 |
| Chad | 110.1 | 106.1 | 96.4 | 4.0 | 3.6 | 0.0 | 0.0 | 0.0 | 0.0 | 0.0 | 0.0 |
| D.R. Congo | 1,263.7 | 428.6 | 33.9 | 672.9 | 53.2 | 6.0 | 0.5 | 89.9 | 7.1 | 66.3 | 5.2 |
| Eq. Guinea | 10.7 | 10.7 | 100.0 | 0.0 | 0.0 | 0.0 | 0.0 | 0.0 | 0.0 | 0.0 | 0.0 |
| Gabon | 30.8 | 30.8 | 100.0 | 0.0 | 0.0 | 0.0 | 0.0 | 0.0 | 0.0 | 0.0 | 0.0 |
| Rep. of Congo | 49.2 | 49.2 | 100.0 | 0.0 | 0.0 | 0.0 | 0.0 | 0.0 | 0.0 | 0.0 | 0.0 |
| **Total CARN** | 2,028.2 | 1,153.0 | 56.9 | 713.0 | 35.2 | 6.0 | 0.3 | 89.9 | 4.4 | 66.3 | 3.3 |
|  |  |  |  |  |  |  |  |  |  |  |  |
| Burundi | 91.7 | 0.0 | 0.0 | 9.4 | 10.3 | 0.0 | 0.0 | 0.6 | 0.7 | 81.7 | 89.1 |
| Djibouti | 1.0 | 0.0 | 0.0 | 1.0 | 100.0 | 0.0 | 0.0 | 0.0 | 0.0 | 0.0 | 0.0 |
| Eritrea | 5.6 | 0.0 | 0.0 | 5.5 | 96.6 | 0.2 | 3.4 | 0.0 | 0.0 | 0.0 | 0.0 |
| Ethiopia | 50.0 | 0.0 | 0.0 | 14.4 | 28.8 | 35.6 | 71.2 | 0.0 | 0.0 | 0.0 | 0.0 |
| Kenya | 149.7 | 0.0 | 0.0 | 3.0 | 2.0 | 116.9 | 78.1 | 24.5 | 16.3 | 5.3 | 3.6 |
| Rwanda | 22.9 | 0.0 | 0.0 | 0.0 | 0.0 | 0.0 | 0.0 | 0.0 | 0.0 | 22.9 | 100.0 |
| Somalia | 24.9 | 0.0 | 0.0 | 24.9 | 100.0 | 0.0 | 0.0 | 0.0 | 0.0 | 0.0 | 0.0 |
| S. Sudan | 80.8 | 0.0 | 0.0 | 61.1 | 75.7 | 19.7 | 24.3 | 0.0 | 0.0 | 0.0 | 0.0 |
| Sudan | 84.7 | 0.0 | 0.0 | 83.8 | 98.9 | 0.9 | 1.1 | 0.0 | 0.0 | 0.0 | 0.0 |
| Uganda | 332.6 | 0.0 | 0.0 | 0.0 | 0.0 | 179.4 | 53.9 | 103.1 | 31.0 | 50.1 | 15.1 |
| **Total EARN** | 844.0 | 0.0 | 0.0 | 203.2 | 24.1 | 352.6 | 41.8 | 128.2 | 15.2 | 160.0 | 19.0 |
|  |  |  |  |  |  |  |  |  |  |  |  |
| Botswana | 2.2 | 0.0 | 0.0 | 2.2 | 100.0 | 0.0 | 0.0 | 0.0 | 0.0 | 0.0 | 0.0 |
| Madagascar | 75.3 | 75.3 | 100.0 | 0.0 | 0.0 | 0.0 | 0.0 | 0.0 | 0.0 | 0.0 | 0.0 |
| Malawi | 165.3 | 0.0 | 0.0 | 0.0 | 0.0 | 165.3 | 100.0 | 0.0 | 0.0 | 0.0 | 0.0 |
| Mozambique | 535.5 | 0.0 | 0.0 | 447.0 | 83.5 | 88.5 | 16.5 | 0.0 | 0.0 | 0.0 | 0.0 |
| Namibia | 8.9 | 6.1 | 68.9 | 2.8 | 31.1 | 0.0 | 0.0 | 0.0 | 0.0 | 0.0 | 0.0 |
| South Africa | 27.0 | 0.0 | 0.0 | 27.0 | 100.0 | 0.0 | 0.0 | 0.0 | 0.0 | 0.0 | 0.0 |
| Swaziland | 0.1 | 0.0 | 0.0 | 0.1 | 100.0 | 0.0 | 0.0 | 0.0 | 0.0 | 0.0 | 0.0 |
| Tanzania | 281.4 | 0.0 | 0.0 | 123.5 | 43.9 | 72.5 | 25.8 | 56.1 | 19.9 | 29.3 | 10.4 |
| Zambia | 147.3 | 0.0 | 0.0 | 102.7 | 69.7 | 44.6 | 30.3 | 0.0 | 0.0 | 0.0 | 0.0 |
| Zimbabwe | 31.0 | 0.0 | 0.0 | 31.0 | 99.9 | 0.0 | 0.1 | 0.0 | 0.0 | 0.0 | 0.0 |
| **Total SARN** | 1,274.0 | 81.4 | 6.4 | 736.2 | 57.8 | 370.9 | 29.1 | 56.1 | 4.4 | 29.3 | 2.3 |
|  |  |  |  |  |  |  |  |  |  |  |  |
| **Total SSA** | 9,470.0 | 6,558.3 | 69.3 | 1,652.4 | 17.4 | 729.5 | 7.7 | 274.2 | 2.9 | 255.6 | 2.7 |

**Table S3-6 Total number of infected primigravidae (1000s) by SP resistance stratum**

|  |  | **Number of infected primigravidae, by SP resistance stratum** | | | | | | | | | |
| --- | --- | --- | --- | --- | --- | --- | --- | --- | --- | --- | --- |
|  | Total No. infected | IPTp-SP fully effective |  | IPTp-SP effective |  | SP at risk (1) |  | SP at risk(2) |  | Super-resistant |  |
|  |  | N | % | N | % | N | % | N | % | N | % |
| Burkina Faso | 88.4 | 88.4 | 100.0 | 0.0 | 0.0 | 0.0 | 0.0 | 0.0 | 0.0 | 0.0 | 0.0 |
| Benin | 37.6 | 37.6 | 100.0 | 0.0 | 0.0 | 0.0 | 0.0 | 0.0 | 0.0 | 0.0 | 0.0 |
| Cote d'Ivoire | 94.3 | 94.3 | 100.0 | 0.0 | 0.0 | 0.0 | 0.0 | 0.0 | 0.0 | 0.0 | 0.0 |
| The Gambia | 1.1 | 1.1 | 100.0 | 0.0 | 0.0 | 0.0 | 0.0 | 0.0 | 0.0 | 0.0 | 0.0 |
| Ghana | 101.1 | 101.1 | 100.0 | 0.0 | 0.0 | 0.0 | 0.0 | 0.0 | 0.0 | 0.0 | 0.0 |
| Guinea | 52.9 | 52.9 | 100.0 | 0.0 | 0.0 | 0.0 | 0.0 | 0.0 | 0.0 | 0.0 | 0.0 |
| Guinea-Bissau | 2.5 | 2.5 | 100.0 | 0.0 | 0.0 | 0.0 | 0.0 | 0.0 | 0.0 | 0.0 | 0.0 |
| Liberia | 14.5 | 14.5 | 100.0 | 0.0 | 0.0 | 0.0 | 0.0 | 0.0 | 0.0 | 0.0 | 0.0 |
| Mali | 67.4 | 67.4 | 100.0 | 0.0 | 0.0 | 0.0 | 0.0 | 0.0 | 0.0 | 0.0 | 0.0 |
| Mauritania | 3.4 | 3.4 | 100.0 | 0.0 | 0.0 | 0.0 | 0.0 | 0.0 | 0.0 | 0.0 | 0.0 |
| Niger | 50.0 | 50.0 | 100.0 | 0.0 | 0.0 | 0.0 | 0.0 | 0.0 | 0.0 | 0.0 | 0.0 |
| Nigeria | 601.6 | 601.6 | 100.0 | 0.0 | 0.0 | 0.0 | 0.0 | 0.0 | 0.0 | 0.0 | 0.0 |
| Sierra Leone | 26.4 | 26.4 | 100.0 | 0.0 | 0.0 | 0.0 | 0.0 | 0.0 | 0.0 | 0.0 | 0.0 |
| Senegal | 21.0 | 21.0 | 100.0 | 0.0 | 0.0 | 0.0 | 0.0 | 0.0 | 0.0 | 0.0 | 0.0 |
| Togo | 37.7 | 37.7 | 100.0 | 0.0 | 0.0 | 0.0 | 0.0 | 0.0 | 0.0 | 0.0 | 0.0 |
| **Total WARN** | 1,199.9 | 1,199.9 | 100.0 | 0.0 | 0.0 | 0.0 | 0.0 | 0.0 | 0.0 | 0.0 | 0.0 |
|  |  |  |  |  |  |  |  |  |  |  |  |
| Angola | 27.3 | 25.2 | 92.3 | 2.1 | 7.7 | 0.0 | 0.0 | 0.0 | 0.0 | 0.0 | 0.0 |
| C. African Rep. | 22.9 | 18.6 | 81.1 | 4.3 | 18.9 | 0.0 | 0.0 | 0.0 | 0.0 | 0.0 | 0.0 |
| Cameroon | 65.2 | 65.2 | 100.0 | 0.0 | 0.0 | 0.0 | 0.0 | 0.0 | 0.0 | 0.0 | 0.0 |
| Chad | 20.2 | 19.5 | 96.6 | 0.7 | 3.4 | 0.0 | 0.0 | 0.0 | 0.0 | 0.0 | 0.0 |
| D.R. Congo | 233.1 | 78.8 | 33.8 | 124.3 | 53.3 | 1.1 | 0.5 | 16.8 | 7.2 | 12.0 | 5.2 |
| Eq. Guinea | 2.6 | 2.6 | 100.0 | 0.0 | 0.0 | 0.0 | 0.0 | 0.0 | 0.0 | 0.0 | 0.0 |
| Gabon | 6.1 | 6.1 | 100.0 | 0.0 | 0.0 | 0.0 | 0.0 | 0.0 | 0.0 | 0.0 | 0.0 |
| Rep. of Congo | 11.1 | 11.1 | 100.0 | 0.0 | 0.0 | 0.0 | 0.0 | 0.0 | 0.0 | 0.0 | 0.0 |
| **Total CARN** | 388.5 | 227.1 | 58.5 | 131.5 | 33.8 | 1.1 | 0.3 | 16.8 | 4.3 | 12.0 | 3.1 |
|  |  |  |  |  |  |  |  |  |  |  |  |
| Burundi | 19.8 | 0.0 | 0.0 | 1.9 | 9.5 | 0.0 | 0.0 | 0.1 | 0.6 | 17.8 | 89.9 |
| Djibouti | 0.4 | 0.0 | 0.0 | 0.4 | 100.0 | 0.0 | 0.0 | 0.0 | 0.0 | 0.0 | 0.0 |
| Eritrea | 1.4 | 0.0 | 0.0 | 1.4 | 97.1 | 0.0 | 2.9 | 0.0 | 0.0 | 0.0 | 0.0 |
| Ethiopia | 12.3 | 0.0 | 0.0 | 3.5 | 28.8 | 8.7 | 71.2 | 0.0 | 0.0 | 0.0 | 0.0 |
| Kenya | 41.7 | 0.0 | 0.0 | 0.8 | 1.9 | 32.5 | 77.9 | 7.1 | 17.1 | 1.3 | 3.1 |
| Rwanda | 6.6 | 0.0 | 0.0 | 0.0 | 0.0 | 0.0 | 0.0 | 0.0 | 0.0 | 6.6 | 100.0 |
| Somalia | 6.0 | 0.0 | 0.0 | 6.0 | 100.0 | 0.0 | 0.0 | 0.0 | 0.0 | 0.0 | 0.0 |
| S. Sudan | 14.8 | 0.0 | 0.0 | 11.1 | 74.9 | 3.7 | 25.1 | 0.0 | 0.0 | 0.0 | 0.0 |
| Sudan | 16.3 | 0.0 | 0.0 | 16.1 | 99.0 | 0.2 | 1.0 | 0.0 | 0.0 | 0.0 | 0.0 |
| Uganda | 71.5 | 0.0 | 0.0 | 0.0 | 0.0 | 38.9 | 54.4 | 21.9 | 30.7 | 10.7 | 15.0 |
| **Total EARN** | 190.8 | 0.0 | 0.0 | 41.2 | 21.6 | 84.0 | 44.0 | 29.2 | 15.3 | 36.4 | 19.1 |
|  |  |  |  |  |  |  |  |  |  |  |  |
| Botswana | 0.6 | 0.0 | 0.0 | 0.6 | 100.0 | 0.0 | 0.0 | 0.0 | 0.0 | 0.0 | 0.0 |
| Madagascar | 19.9 | 19.9 | 100.0 | 0.0 | 0.0 | 0.0 | 0.0 | 0.0 | 0.0 | 0.0 | 0.0 |
| Malawi | 39.1 | 0.0 | 0.0 | 0.0 | 0.0 | 39.1 | 100.0 | 0.0 | 0.0 | 0.0 | 0.0 |
| Mozambique | 99.4 | 0.0 | 0.0 | 83.1 | 83.7 | 16.2 | 16.3 | 0.0 | 0.0 | 0.0 | 0.0 |
| Namibia | 2.3 | 1.6 | 70.2 | 0.7 | 29.8 | 0.0 | 0.0 | 0.0 | 0.0 | 0.0 | 0.0 |
| South Africa | 6.0 | 0.0 | 0.0 | 6.0 | 100.0 | 0.0 | 0.0 | 0.0 | 0.0 | 0.0 | 0.0 |
| Swaziland | 0.0 | 0.0 | 0.0 | 0.0 | 100.0 | 0.0 | 0.0 | 0.0 | 0.0 | 0.0 | 0.0 |
| Tanzania | 60.0 | 0.0 | 0.0 | 26.8 | 44.6 | 15.2 | 25.4 | 11.9 | 19.8 | 6.1 | 10.2 |
| Zambia | 30.5 | 0.0 | 0.0 | 21.5 | 70.7 | 8.9 | 29.3 | 0.0 | 0.0 | 0.0 | 0.0 |
| Zimbabwe | 7.6 | 0.0 | 0.0 | 7.6 | 99.9 | 0.0 | 0.1 | 0.0 | 0.0 | 0.0 | 0.0 |
| **Total SARN** | 265.3 | 21.5 | 8.1 | 146.3 | 55.2 | 79.5 | 30.0 | 11.9 | 4.5 | 6.1 | 2.3 |
|  |  |  |  |  |  |  |  |  |  |  |  |
| **Total SSA** | 2,044.5 | 1,448.5 | 70.8 | 319.0 | 15.6 | 164.6 | 8.0 | 57.9 | 2.8 | 54.6 | 2.7 |

**Table S3-7 Total number of malaria attributable low birthweight (LBW) deliveries**

|  |  | **Number of malaria attributable low birth weight deliveries, by SP resistance stratum** | | | | | | | | | |
| --- | --- | --- | --- | --- | --- | --- | --- | --- | --- | --- | --- |
|  | Total No. MA-LBW deliveries | IPTp-SP fully effective |  | IPTp-SP effective |  | SP at risk (1) |  | SP at risk(2) |  | Super-resistant |  |
|  |  | N | % | N | % | N | % | N | % | N | % |
| Burkina Faso | 29,422 | 29,422 | 100 | 0 | 0 | 0 | 0 | 0 | 0 | 0 | 0 |
| Benin | 13,040 | 13,040 | 100 | 0 | 0 | 0 | 0 | 0 | 0 | 0 | 0 |
| Cote d'Ivoire | 28,987 | 28,987 | 100 | 0 | 0 | 0 | 0 | 0 | 0 | 0 | 0 |
| The Gambia | 563 | 563 | 100 | 0 | 0 | 0 | 0 | 0 | 0 | 0 | 0 |
| Ghana | 30,343 | 30,343 | 100 | 0 | 0 | 0 | 0 | 0 | 0 | 0 | 0 |
| Guinea | 16,994 | 16,994 | 100 | 0 | 0 | 0 | 0 | 0 | 0 | 0 | 0 |
| Guinea-Bissau | 1,051 | 1,051 | 100 | 0 | 0 | 0 | 0 | 0 | 0 | 0 | 0 |
| Liberia | 5,112 | 5,112 | 100 | 0 | 0 | 0 | 0 | 0 | 0 | 0 | 0 |
| Mali | 23,165 | 23,165 | 100 | 0 | 0 | 0 | 0 | 0 | 0 | 0 | 0 |
| Mauritania | 1,791 | 1,791 | 100 | 0 | 0 | 0 | 0 | 0 | 0 | 0 | 0 |
| Niger | 21,092 | 21,092 | 100 | 0 | 0 | 0 | 0 | 0 | 0 | 0 | 0 |
| Nigeria | 210,281 | 210,281 | 100 | 0 | 0 | 0 | 0 | 0 | 0 | 0 | 0 |
| Sierra Leone | 8,751 | 8,751 | 100 | 0 | 0 | 0 | 0 | 0 | 0 | 0 | 0 |
| Senegal | 8,839 | 8,839 | 100 | 0 | 0 | 0 | 0 | 0 | 0 | 0 | 0 |
| Togo | 12,306 | 12,306 | 100 | 0 | 0 | 0 | 0 | 0 | 0 | 0 | 0 |
| **Total WARN** | 411,737 | 411,737 | 100 | 0 | 0 | 0 | 0 | 0 | 0 | 0 | 0 |
|  |  |  |  |  |  |  |  |  |  |  |  |
| Angola | 12,807 | 11,818 | 92.3 | 989 | 7.7 | 0 | 0 | 0 | 0 | 0 | 0 |
| C. African Rep. | 8,302 | 6,718 | 80.9 | 1,584 | 19.1 | 0 | 0 | 0 | 0 | 0 | 0 |
| Cameroon | 23,498 | 23,498 | 100 | 0 | 0 | 0 | 0 | 0 | 0 | 0 | 0 |
| Chad | 9,620 | 9,252 | 96.2 | 368 | 3.8 | 0 | 0 | 0 | 0 | 0 | 0 |
| D.R. Congo | 92,736 | 33,024 | 35.6 | 47,568 | 51.3 | 391 | 0.4 | 6,192 | 6.7 | 5,561 | 6.0 |
| Eq. Guinea | 842 | 842 | 100 | 0 | 0 | 0 | 0 | 0 | 0 | 0 | 0 |
| Gabon | 2,374 | 2,374 | 100 | 0 | 0 | 0 | 0 | 0 | 0 | 0 | 0 |
| Rep. of Congo | 4,342 | 4,342 | 100 | 0 | 0 | 0 | 0 | 0 | 0 | 0 | 0 |
| **Total CARN** | 154,522 | 91,869 | 59.5 | 50,509 | 32.7 | 391 | 0.3 | 6,192 | 4.0 | 5,561 | 3.6 |
|  |  |  |  |  |  |  |  |  |  |  |  |
| Burundi | 8,192 | 0 | 0 | 866 | 10.6 | 0 | 0 | 51 | 0.6 | 7,275 | 88.8 |
| Djibouti | 106 | 0 | 0 | 106 | 100 | 0 | 0 | 0 | 0 | 0 | 0 |
| Eritrea | 578 | 0 | 0 | 559 | 96.7 | 19 | 3.3 | 0 | 0 | 0 | 0 |
| Ethiopia | 5,216 | 0 | 0 | 1,504 | 28.8 | 3,712 | 71.2 | 0 | 0 | 0 | 0 |
| Kenya | 14,624 | 0 | 0 | 288 | 2.0 | 11,555 | 79.0 | 2,302 | 15.7 | 479 | 3.3 |
| Rwanda | 2,332 | 0 | 0 | 0 | 0 | 0 | 0 | 0 | 0 | 2,332 | 100 |
| Somalia | 2,461 | 0 | 0 | 2,460 | 100 | 1 | 0 | 0 | 0 | 0 | 0 |
| S. Sudan | 6,681 | 0 | 0 | 5,129 | 76.8 | 1,552 | 23.2 | 0 | 0 | 0 | 0 |
| Sudan | 8,149 | 0 | 0 | 8,068 | 99.0 | 81 | 1.0 | 0 | 0 | 0 | 0 |
| Uganda | 29,042 | 0 | 0 | 0 | 0 | 15,556 | 53.6 | 8,921 | 30.7 | 4,565 | 15.7 |
| **Total EARN** | 77,381 | 0 | 0 | 18,980 | 24.5 | 32,476 | 42.0 | 11,274 | 14.6 | 14,651 | 18.9 |
|  |  |  |  |  |  |  |  |  |  |  |  |
| Botswana | 223 | 0 | 0 | 223 | 100 | 0 | 0 | 0 | 0 | 0 | 0 |
| Madagascar | 7,340 | 7,340 | 100 | 0 | 0 | 0 | 0 | 0 | 0 | 0 | 0 |
| Malawi | 14,665 | 0 | 0 | 0 | 0 | 14,665 | 100 | 0 | 0 | 0 | 0 |
| Mozambique | 38,568 | 0 | 0 | 31,976 | 82.9 | 6,592 | 17.1 | 0 | 0 | 0 | 0 |
| Namibia | 864 | 599 | 69.3 | 265 | 30.7 | 0 | 0 | 0 | 0 | 0 | 0 |
| South Africa | 2,589 | 0 | 0 | 2,589 | 100 | 0 | 0 | 0 | 0 | 0 | 0 |
| Swaziland | 6 | 0 | 0 | 6 | 100 | 0 | 0 | 0 | 0 | 0 | 0 |
| Tanzania | 25,717 | 0 | 0 | 11,171 | 43.4 | 6,849 | 26.6 | 5,011 | 19.5 | 2,686 | 10.4 |
| Zambia | 12,067 | 0 | 0 | 8,629 | 71.5 | 3,438 | 28.5 | 0 | 0 | 0 | 0 |
| Zimbabwe | 3,102 | 0 | 0 | 3,100 | 99.9 | 2 | 0.1 | 0 | 0 | 0 | 0 |
| **Total SARN** | 105,140 | 7,939 | 7.6 | 57,959 | 55.1 | 31,546 | 30 | 5,011 | 4.8 | 2,686 | 2.6 |
|  |  |  |  |  |  |  |  |  |  |  |  |
| **Total SSA** | 748,781 | 511,546 | 68.3 | 127,448 | 17.0 | 64,412 | 8.6 | 22,477 | 3.0 | 22,897 | 3.1 |

**Table S3-8 Total number of malaria attributable LBW deliveries in primigravidae**

|  |  | **Number of malaria attributable low birth weight deliveries, by SP resistance stratum** | | | | | | | | | |
| --- | --- | --- | --- | --- | --- | --- | --- | --- | --- | --- | --- |
|  | Total No. MA-LBW in PG | IPTp-SP fully effective |  | IPTp-SP effective |  | SP at risk (1) |  | SP at risk(2) |  | Super-resistant |  |
|  |  | N | % | N | % | N | % | N | % | N | % |
| Burkina Faso | 11,597 | 11,597 | 100 | 0 | 0 | 0 | 0 | 0 | 0 | 0 | 0 |
| Benin | 4,613 | 4,613 | 100 | 0 | 0 | 0 | 0 | 0 | 0 | 0 | 0 |
| Cote d'Ivoire | 11,968 | 11,968 | 100 | 0 | 0 | 0 | 0 | 0 | 0 | 0 | 0 |
| The Gambia | 124 | 124 | 100 | 0 | 0 | 0 | 0 | 0 | 0 | 0 | 0 |
| Ghana | 12,460 | 12,460 | 100 | 0 | 0 | 0 | 0 | 0 | 0 | 0 | 0 |
| Guinea | 7,031 | 7,031 | 100 | 0 | 0 | 0 | 0 | 0 | 0 | 0 | 0 |
| Guinea-Bissau | 275 | 275 | 100 | 0 | 0 | 0 | 0 | 0 | 0 | 0 | 0 |
| Liberia | 1,775 | 1,775 | 100 | 0 | 0 | 0 | 0 | 0 | 0 | 0 | 0 |
| Mali | 9,069 | 9,069 | 100 | 0 | 0 | 0 | 0 | 0 | 0 | 0 | 0 |
| Mauritania | 372 | 372 | 100 | 0 | 0 | 0 | 0 | 0 | 0 | 0 | 0 |
| Niger | 6,018 | 6,018 | 100 | 0 | 0 | 0 | 0 | 0 | 0 | 0 | 0 |
| Nigeria | 74,265 | 74,265 | 100 | 0 | 0 | 0 | 0 | 0 | 0 | 0 | 0 |
| Sierra Leone | 3,240 | 3,240 | 100 | 0 | 0 | 0 | 0 | 0 | 0 | 0 | 0 |
| Senegal | 2,338 | 2,338 | 100 | 0 | 0 | 0 | 0 | 0 | 0 | 0 | 0 |
| Togo | 5,002 | 5,002 | 100 | 0 | 0 | 0 | 0 | 0 | 0 | 0 | 0 |
| **Total WARN** | 150,146 | 150,146 | 100 | 0 | 0 | 0 | 0 | 0 | 0 | 0 | 0 |
|  |  |  |  |  |  |  |  |  |  |  |  |
| Angola | 3,147 | 2,902 | 92.2 | 245 | 7.8 | 0 | 0 | 0 | 0 | 0 | 0 |
| C. African Rep. | 2,841 | 2,291 | 80.6 | 550 | 19.4 | 0 | 0 | 0 | 0 | 0 | 0 |
| Cameroon | 7,815 | 7,815 | 100 | 0 | 0 | 0 | 0 | 0 | 0 | 0 | 0 |
| Chad | 2,273 | 2,197 | 96.7 | 76 | 3.3 | 0 | 0 | 0 | 0 | 0 | 0 |
| D.R. Congo | 28,397 | 9,335 | 32.9 | 15,444 | 54.4 | 139 | 0.5 | 2,106 | 7.4 | 1,372 | 4.8 |
| Eq. Guinea | 313 | 313 | 100 | 0 | 0 | 0 | 0 | 0 | 0 | 0 | 0 |
| Gabon | 730 | 730 | 100 | 0 | 0 | 0 | 0 | 0 | 0 | 0 | 0 |
| Rep. of Congo | 1,281 | 1,281 | 100 | 0 | 0 | 0 | 0 | 0 | 0 | 0 | 0 |
| **Total CARN** | 46,798 | 26,865 | 57.4 | 16,316 | 34.9 | 139 | 0.3 | 2,106 | 4.5 | 1,372 | 2.9 |
|  |  |  |  |  |  |  |  |  |  |  |  |
| Burundi | 2,248 | 0 | 0 | 210 | 9.3 | 0 | 0 | 13 | 0.6 | 2,025 | 90.1 |
| Djibouti | 45 | 0 | 0 | 45 | 100 | 0 | 0 | 0 | 0 | 0 | 0 |
| Eritrea | 153 | 0 | 0 | 148 | 96.7 | 4 | 2.6 | 0 | 0 | 0 | 0 |
| Ethiopia | 1,310 | 0 | 0 | 377 | 28.8 | 933 | 71.2 | 0 | 0 | 0 | 0 |
| Kenya | 4,647 | 0 | 0 | 89 | 1.9 | 3,589 | 77.2 | 818 | 17.6 | 151 | 3.2 |
| Rwanda | 720 | 0 | 0 | 0 | 0 | 0 | 0 | 0 | 0 | 720 | 100 |
| Somalia | 654 | 0 | 0 | 654 | 100 | 0 | 0 | 0 | 0 | 0 | 0 |
| S. Sudan | 1,694 | 0 | 0 | 1,260 | 74.4 | 434 | 25.6 | 0 | 0 | 0 | 0 |
| Sudan | 1,769 | 0 | 0 | 1,751 | 99.0 | 18 | 1.0 | 0 | 0 | 0 | 0 |
| Uganda | 8,195 | 0 | 0 | 0 | 0 | 4,478 | 54.6 | 2,513 | 30.7 | 1,204 | 14.7 |
| **Total EARN** | 21,434 | 0 | 0 | 4,535 | 21.2 | 9,456 | 44.1 | 3,343 | 15.6 | 4,099 | 19.1 |
|  |  |  |  |  |  |  |  |  |  |  |  |
| Botswana | 60 | 0 | 0 | 60 | 100 | 0 | 0 | 0 | 0 | 0 | 0 |
| Madagascar | 2,209 | 2,209 | 100 | 0 | 0 | 0 | 0 | 0 | 0 | 0 | 0 |
| Malawi | 4,557 | 0 | 0 | 0 | 0 | 4,557 | 100 | 0 | 0 | 0 | 0 |
| Mozambique | 12,347 | 0 | 0 | 10,372 | 84.0 | 1,975 | 16.0 | 0 | 0 | 0 | 0 |
| Namibia | 253 | 177 | 70 | 76 | 30 | 0 | 0 | 0 | 0 | 0 | 0 |
| South Africa | 659 | 0 | 0 | 659 | 100 | 0 | 0 | 0 | 0 | 0 | 0 |
| Swaziland | 2 | 0 | 0 | 2 | 100 | 0 | 0 | 0 | 0 | 0 | 0 |
| Tanzania | 6,722 | 0 | 0 | 3,012 | 44.8 | 1,684 | 25.1 | 1,341 | 19.9 | 686 | 10.2 |
| Zambia | 3,583 | 0 | 0 | 2,505 | 69.9 | 1,078 | 30.1 | 0 | 0 | 0 | 0 |
| Zimbabwe | 835 | 0 | 0 | 835 | 100 | 1 | 0.1 | 0 | 0 | 0 | 0 |
| **Total SARN** | 31,228 | 2,386 | 7.6 | 17,521 | 56.1 | 9,295 | 29.8 | 1,341 | 4.3 | 686 | 2.2 |
|  |  |  |  |  |  |  |  |  |  |  |  |
| **Total SSA** | 249,606 | 179,397 | 71.9 | 38,371 | 15.4 | 18,891 | 7.6 | 6,791 | 2.7 | 6,156 | 2.5 |
